# Bayesian multi-source regression and monocyte-associated gene expression predict BCL-2 inhibitor resistance in acute myeloid leukemia

**DOI:** 10.1038/s41698-021-00209-9

**Published:** 2021-07-23

**Authors:** Brian S. White, Suleiman A. Khan, Mike J. Mason, Muhammad Ammad-ud-din, Swapnil Potdar, Disha Malani, Heikki Kuusanmäki, Brian J. Druker, Caroline Heckman, Olli Kallioniemi, Stephen E. Kurtz, Kimmo Porkka, Cristina E. Tognon, Jeffrey W. Tyner, Tero Aittokallio, Krister Wennerberg, Justin Guinney

**Affiliations:** 1 Computational Oncology, Sage Bionetworks, Seattle, WA USA; 2 Institute for Molecular Medicine Finland (FIMM), Helsinki Institute of Life Science (HiLIFE), University of Helsinki, Helsinki, Finland; 3 Biotech Research & Innovation Centre (BRIC) and Novo Nordisk Foundation Center for Stem Cell Biology (DanStem), University of Copenhagen, Copenhagen, Denmark; 4 Howard Hughes Medical Institute, Portland, OR USA; 5 Division of Hematology and Medical Oncology, Knight Cancer Institute, Oregon Health & Science University, Portland, OR USA; 6 Scilifelab, Karolinska Institute, Solna, Sweden; 7 HUS Comprehensive Cancer Center, Hematology Research Unit Helsinki and iCAN Digital Precision Cancer Center Medicine Flagship, University of Helsinki, Helsinki, Finland; 8 Department of Mathematics and Statistics, University of Turku, Turku, Finland; 9 Department of Cancer Genetics, Institute for Cancer Research, Oslo University Hospital, Oslo, Norway; 10 Centre for Biostatistics and Epidemiology (OCBE), University of Oslo, Oslo, Norway; 11 Department of Biomedical Informatics and Medical Education, University of Washington, Seattle, WA USA; 12 Present Address: The Jackson Laboratory for Genomic Medicine, Farmington, CT USA

**Keywords:** High-throughput screening, Acute myeloid leukaemia, Cancer genomics, Gene expression analysis, Predictive markers

## Abstract

The FDA recently approved eight targeted therapies for acute myeloid leukemia (AML), including the BCL-2 inhibitor venetoclax. Maximizing efficacy of these treatments requires refining patient selection. To this end, we analyzed two recent AML studies profiling the gene expression and ex vivo drug response of primary patient samples. We find that ex vivo samples often exhibit a general sensitivity to (any) drug exposure, independent of drug target. We observe that this “general response across drugs” (GRD) is associated with *FLT3*-ITD mutations, clinical response to standard induction chemotherapy, and overall survival. Further, incorporating GRD into expression-based regression models trained on one of the studies improved their performance in predicting ex vivo response in the second study, thus signifying its relevance to precision oncology efforts. We find that venetoclax response is independent of GRD but instead show that it is linked to expression of monocyte-associated genes by developing and applying a multi-source Bayesian regression approach. The method shares information across studies to robustly identify biomarkers of drug response and is broadly applicable in integrative analyses.

## Introduction

Acute myeloid leukemia (AML) is genetically, epigenetically, and transcriptionally heterogeneous. Nevertheless, patient treatment has been uniform and for decades standard first-line treatment has been “7 + 3” induction chemotherapy with cytarabine and an anthracycline. In addition, a significant portion of AML patients are not considered fit enough to tolerate induction chemotherapy and are instead typically treated with low-dose cytarabine (LDAC) and hypomethylating agents (HMAs), e.g., azacitidine or decitabine, that extend survival but are rarely curative. Together, outcomes for adult AML patients (aged ≥20 years) remain very poor, with the current 5-year relative survival rate at 24%^1^. The US Food and Drug Administration (FDA) has recently shifted this therapeutic landscape by approving eight drugs (enasidenib, gemtuzumab ozogamicin, glasdegib, ivosidenib, midostaurin, gilteritinib, venetoclax, and a liposome-encapsulated combination of daunorubicin and cytarabine) for newly diagnosed or relapse refractory patients, alone or in combination with LDAC or HMAs^2,3^. Maximizing clinical benefit from these treatments will require biomarkers for optimized patient selection and rational drug combination strategies.

We used data from two independent studies that comprehensively profiled AML patient cohorts to address this need. In both studies, ex vivo functional drug testing was performed on freshly isolated mononuclear cells from AML patients and cell viability was assessed following drug exposure across a concentration range. The multi-center Beat AML initiative, led by Oregon Health & Science University (OHSU), profiled 562 patient samples, including 411 with RNA sequencing (RNA-seq), 531 with exome sequencing, and 363 across a panel of 122 small-molecule inhibitors^4^. The AML Individualized Systems Medicine program at the Institute for Molecular Medicine Finland (FIMM) profiled 37 patients using RNA-seq, exome sequencing, and across a panel of 470 inhibitors^5^. We performed a comparative analysis, training regression models in one study to predict ex vivo drug response in the other. In both studies, we observed a tendency of a patient-derived sample to respond consistently across all drugs. This “general response across drugs” (GRD) correlated with complete response to standard induction therapy and with patient leukemia-free survival. Including GRD in the models significantly improved their performance.

Several drugs did not conform to this trend, including the BCL-2 inhibitor venetoclax^6^, which was recently approved in combination with HMAs or LDAC for the treatment of AML in newly diagnosed elderly patients or in those unfit for intensive chemotherapy. Venetoclax in combination with azacitidine or decitabine showed a favorable overall response rate [complete remission (CR) + CR with incomplete count recovery (CRi)] of 67% in a phase 1b study of 145 patients aged ≥65 years with newly diagnosed AML^7^, whereas an overall response rate of 28% has been observed for a similar patient population treated with azacitidine alone^8^. Nevertheless, a large minority of patients do not respond to venetoclax, while the majority who do ultimately relapse^7^.

As such, recent efforts have attempted to refine patient selection for venetoclax treatment. An ex vivo study^9^ suggested that patients with monocytic AML have reduced sensitivity to venetoclax. An in vivo study^10^ additionally demonstrated that intra-patient heterogeneity arising from monocytic subclones contributes to venetoclax resistance. Here we show that the degree of patient monocyticity accounts for the majority of inter-patient variation in resistance. We quantify this effect through a robust signature comprised of monocyte-associated genes identified via a Bayesian multi-source regression (BMSR) method. BMSR nominates drug biomarkers by performing a joint multi-source analysis across the OHSU and FIMM datasets. The method is broadly applicable in integrating multiple expression datasets to overcome technical variation, biological heterogeneity, and small sample size that contribute to the low reproducibility of biomarker studies^11^.

We extend BMSR to additionally perform simultaneous multi-task analysis across multiple drugs to identify their common biomarkers. Applying it to three mitogen-activated protein kinase kinase (MEK) inhibitors (trametinib, PD184352, and selumetinib) reveals that their response is correlated with the monocytic venetoclax resistance signature. This, coupled with the observation that venetoclax treatment selects for pre-existing monocytic subclones^10^, provides independent rationale for combination therapies targeting the BCL-2 and MEK pathways^12^.

## Results

### GRD is associated with improved patient outcome

**Fig. 1:**
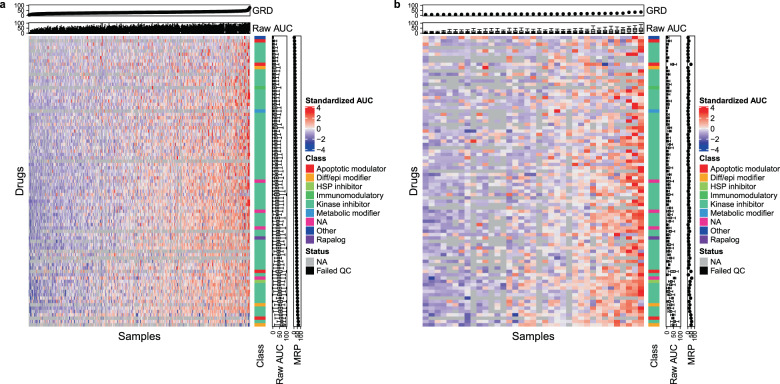
A patient’s ex vivo responses are similar across common drugs. AUCs calculated across patient-derived ex vivo samples (columns) and 87 drugs (rows) common to OHSU (**a**; *n* = 338) and FIMM (**b**; *n* = 37) datasets. Red values correspond to higher AUC or more sensitive samples, blue are less sensitive, black are filtered, and gray are missing. Standardized AUCs (i.e., with mean zero and standard deviation one across patients) displayed in heatmap. Raw AUCs displayed in top and side panels. General response across drugs (GRD) is mean of raw AUCs for an individual patient over drugs; mean response across patients (MRP) is mean of raw AUCs for an individual drug over patients. Samples ordered by GRD in each dataset. Drugs ordered by MRP in OHSU dataset. One sample displayed per patient, with sample assayed across the highest number of drugs displayed in cases with multiple samples per patient. Class drug class, Diff/epi differentiation/epigenetic, HSP heat shock protein.

The 122 OHSU drug panel and the 470 FIMM drug panel shared 87 drugs (71 of which are kinase inhibitors). We used these common drugs to assess the consistency of drug response across the two studies. We first quantified response as area under the dose–response curve (AUC; Supplementary Figs. 1 and 2, Supplementary Table 1, and Fig. 1). Responses were positively correlated across drugs in each dataset [OHSU: 84% of Pearson correlation *r*s are positive, mean *r* = 0.22, 95% confidence interval (CI) = −0.22 to 0.59; FIMM: 85% positive *r*, mean *r* = 0.33, 95% CI = −0.29 to 0.83; Supplementary Fig. 3]. We measured the cross-study correlation of the intra-study drug–drug correlations, i.e., the “correlation of correlations,” a general measure of interstudy consistency (*r* = 0.35, *p* < 10^−10^; Supplementary Fig. 3). As expected, correlations were higher when restricted to drugs with a common target, including for 5 cyclin-dependent kinase inhibitors (*r* = 0.76), 5 epidermal growth factor receptor inhibitors (*r* = 0.51), 7 vascular endothelial growth factor receptor inhibitors (*r* = 0.61), 6 fms-like tyrosine kinase 3 (FLT3) inhibitors (*r* = 0.84), and 4 mammalian target of rapamycin/phosphoinositide 3-kinase inhibitors (*r* = 0.95). We also calculated each drug’s mean response across patients and found these to be highly concordant between the datasets (*r* = 0.67; *p* = 2.04 × 10^−12^; Supplementary Fig. 4). Finally, we assessed the consistency of each individual drug’s response across the two datasets. To do so, we represented each drug in each dataset by a vector of its correlations to all other drugs. For each drug, we then calculated the correlation between these dataset- and drug-specific correlation vectors (Supplementary Fig. 5). This cross-dataset drug correlation was positively associated with the drug’s range of response [i.e., interquartile range (IQR) of unnormalized AUCs] in both the OHSU (*r* = 0.45; *p* = 1.02 × 10^−5^) and FIMM (*r* = 0.47; *p* = 4.55 × 10^−6^) datasets. We found no evidence that drug correlation was associated with its class (analysis of variance *p* = 0.37).

Each patient sample tended to respond uniformly across all drugs in the panel—e.g., many responded relatively poorly to all drugs (blue; leftmost samples, Fig. 1), while a smaller number responded relatively strongly to most drugs (red; rightmost samples, Fig. 1). We describe this phenomenon as a sample’s GRD, i.e., its mean AUC across all drugs. Notably, this trend held also across the full drug panels of each dataset (OHSU: 122 drugs; FIMM: 470 drugs; Supplementary Fig. 6), which are less biased toward tyrosine kinase inhibitors (TKIs) than the shared set of drugs. More specifically, we found that GRD computed across all drugs in a dataset was strongly correlated (*r* > 0.9) with that computed from the common set of drugs, the set of drugs that excludes class III TKIs (of which *FLT3* inhibitors are members), and the set of drugs that excludes all TKIs (Supplementary Fig. 7). Consistently, we found that GRD calculated from a random selection of drugs was highly concordant with GRD calculated from all drugs in the respective dataset for both the OHSU (mean *r* across 100 bootstrap samples = 0.97; 95% CI = 0.95–0.98) and FIMM (mean *r* = 0.99; 95% CI = 0.98–1.00) datasets. An observation similar to GRD, General Levels of Drug Sensitivity (GLDS), has previously been reported across cell lines representing various cancer types^13^.

**Fig. 2:**
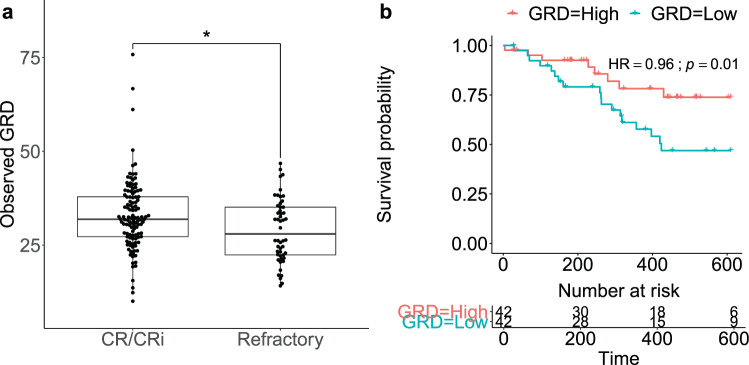
Ex vivo general response across drugs is associated with clinical response and improved patient outcome. **a** GRD in patients who achieve complete remission (CR) or complete remission with incomplete hematologic recovery (CRi) to standard induction chemotherapy (*n* = 118) versus those refractory to induction (*n* = 50) in OHSU dataset. *Wilcoxon rank-sum test *p* < 0.05. **b** Kaplan–Meier survival curves of patients in OHSU dataset with GRD above the upper quartile (red; “responders”; *n* = 42) and of those with GRD below the lower quartile (blue; “non-responders”; *n* = 42). Data are right censored at 610 days. HR: Cox proportional hazard ratio. **a**, **b** GRD is computed across drugs common to OHSU and FIMM datasets.

GRD was higher in samples from patients who achieved a CR or a CRi to standard induction chemotherapy relative to those refractory to induction (two-sided Wilcoxon rank-sum test *p* = 0.02; Fig. 2a; GRD computed across common drugs). Consistently, CR/CRi patients were enriched among those with high GRD (enrichment *p* = 7.5 × 10^−3^; Supplementary Fig. 8). Notably, patients in the top quartile of GRD showed improved overall survival relative to those in the bottom quartile [Cox proportional hazard ratio (HR) = 0.96, log-rank *p* = 0.01; Fig. 2b and Supplementary Fig. 9]. These findings held when GRD was instead computed across all drugs (two-sided Wilcoxon rank-sum test *p* = 8.2 × 10^−4^; HR = 0.95; log-rank *p* = 0.01; Supplementary Fig. 10).

Despite these associations, refractory patients were not statistically enriched in extreme GRD values (enrichment *p* = 0.15; Supplementary Fig. 8). To understand this heterogeneity in response, we examined clinical features, genes, and pathways that differentiated refractory from CR/CRi patients among those with low (bottom quartile) GRD. Increased age was associated with refractory response among GRD-low patients (two-sided Wilcoxon rank-sum test *p* = 9.0 × 10^−3^), though the trend was not significant after correcting for testing of the multiple clinical variables [Benjamini–Hochberg (BH)-adjusted *p* = 0.26; Supplementary Table 2]. One hundred and twenty-two genes were differentially expressed (false discovery rate (FDR) < 20%; Supplementary Tables 3 and 4), with lymphocyte costimulation, T cell receptor signaling, antigen binding, and antigen receptor-mediated signaling (all significant at an FDR < 20%) upregulated in refractory patients and among the strongest enrichments (Supplementary Table 5). Conversely, in examining GRD-high patients, we found that higher creatinine levels trended with CR/CRi (two-sided Wilcoxon rank-sum test *p* = 0.02; BH-adjusted *p* = 0.62; Supplementary Table 6). Forty-six differentially expressed genes (FDR < 20%; Supplementary Table 7) were most strongly enriched within extracellular pathways upregulated in CR/CRi patients (Supplementary Table 8).

### GRD is associated with *FLT3*-ITD

Presence of internal tandem duplication in *FLT3* (*FLT3*-ITD) was positively associated with GRD in the OHSU dataset (BH-adjusted two-sided Wilcoxon rank-sum test *p* = 6.86 × 10^−8^; Supplementary Table 9). This positive association remained significant and independent of *NPM1* mutation status, ethnicity, age, and sex in a multivariate analysis [*R*^2^ = 0.26; *F*-statistic *p* = 6.97 × 10^−10^; two-sided *t* test *p* = 4.95 × 10^−8^; Supplementary Fig. 11 and Supplementary Table 10). We validated the *FLT3*-ITD/GRD association in an independent AML dataset profiling expression and drug response of ex vivo samples published by Tavor and colleagues^14^ (one-sided Wilcoxon rank-sum test *p* < 0.01; Supplementary Fig. 12). We also observed a consistent trend in the FIMM dataset (one-sided Wilcoxon rank-sum test *p* = 0.08). Significantly, we found that *FLT3*-ITD status remained associated with GRD even when the latter was computed from a subset of drugs that excluded *FLT3* inhibitors (Supplementary Fig. 13).

**Fig. 3:**
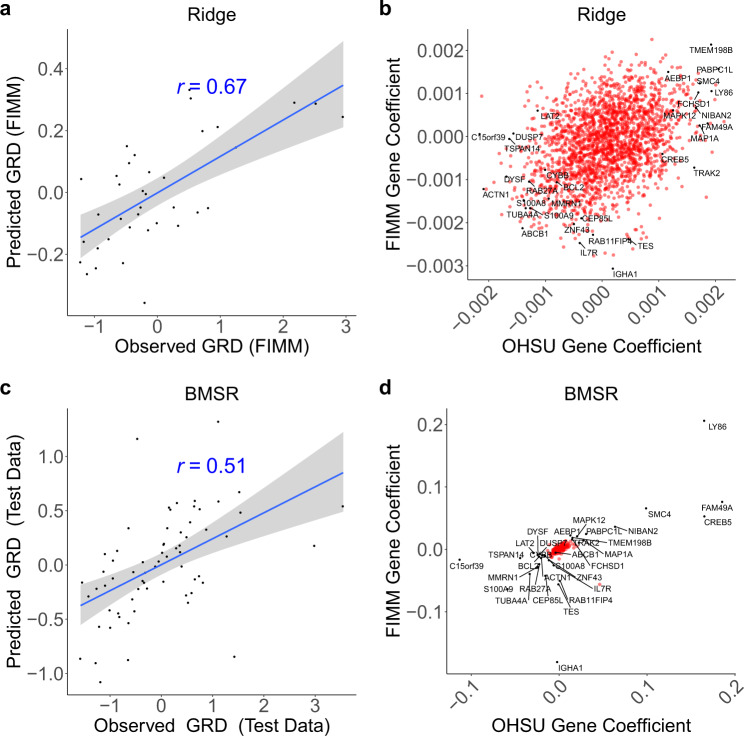
Integrative analysis reveals growth-, apoptosis-, and drug efflux-associated biomarkers of general response across drugs. **a**, **c** Observed (*x* axis) versus model-predicted (*y* axis) GRD. **a** Expression-based ridge regression model trained on OHSU samples (*n* = 292) and tested on FIMM samples (*n* = 37). **c** Expression-based Bayesian multi-source regression (BMSR) model trained using fivefold cross-validation on combined OHSU and FIMM datasets (*n* = 263) and tested on held-out fold yielding median performance across the fivefolds (*n* = 66). **b**, **d** Coefficients of genes (*n* = 2132) in OHSU (*x* axis) or FIMM (*y* axis) datasets following **b** training of ridge regression model independently on both datasets or **d** training of BMSR model simultaneously on entirety of both datasets (*n* = 329). GRD is computed across drugs common to OHSU and FIMM datasets. *r*: Pearson correlation; dashed line: identity line; blue line: linear regression fit; gray shading: 95% confidence interval. Labeled genes were those having extremal (Stouffer’s *p* < 0.01) combined coefficients across both datasets, as well as *ABCB1* (i.e., *MDR1*).

To determine whether GRD could be modeled using gene expression data, we trained an expression-based ridge regression model of GRD using the OHSU dataset (Supplementary Figs. 14–19; Supplementary Table 3; see “Methods”). The model was validated in the FIMM dataset, demonstrating good predictive performance (*r* = 0.67; *p* = 5.6 × 10^−6^; Fig. 3a). We hypothesized that robust biomarkers of GRD should be consistent between the OHSU and FIMM datasets (e.g., having large positive model coefficients in both datasets relative to other genes). To test this, we trained a GRD model on the FIMM dataset and compared the model coefficients associated with each gene between the OHSU- and FIMM-trained models. Unexpectedly, this did not reveal candidate biomarkers with outlying coefficients in both datasets (Fig. 3b). Nevertheless, we did confirm that the *ABCB1* gene [i.e., Multidrug Resistance Protein 1 (*MDR1*)], encoding a drug efflux pump and previously observed to be associated with GLDS^13^, was negatively correlated with GRD in both datasets (Fig. 3b). Further, the ABC transporter family was among the gene sets and pathways having the strongest negative association with GRD (Supplementary Tables 11–15).

### BMSR identifies biomarkers jointly across studies

We reasoned that our inability to detect consistent biomarkers through independent analysis of the two datasets resulted from the highly correlated expression of subsets of genes (e.g., within a pathway), thereby dampening the effect of any single gene and hindering the identification of genes significantly impacting response. This is further complicated by study site-specific technical artifacts, biological variation, and small sample sizes, all of which compound noise. These factors could be ameliorated by performing regularization across multiple datasets simultaneously, which would shrink the coefficients of all but one or a few of the highly correlated genes toward zero.

To do so, we developed an integrative BMSR method that detects consistent and robust biomarkers through joint analysis of the two datasets. BMSR assumes that the biomarker expression/drug response relationship is similar across multiple datasets but allows for relatively small differences due to technical noise, biological variation, or clinical heterogeneity of different patient populations. It achieves this by modeling the contribution to the response by gene *g* in dataset *d* (i.e., the regression coefficient ${\beta }_{g}^{(d)}$) as arising from a mean contribution (i.e., *β*_*g*_) for that gene that is shared across datasets (see “Methods”). BMSR effectively performs regularization on the shared *β*_*g*_ rather than the dataset-specific coefficient ${\beta }_{g}^{(d)}$ through a prior distribution, which acts as a penalty term in a non-Bayesian/frequentist formulation. For two genes *g*_1_ and *g*_2_ that are correlated with response, this approach encourages (but does not guarantee) that the shared coefficient of one of them be shrunk to zero by exploiting correlations in the data rather than explicit biological annotations (e.g., pathways).

Modeling GRD with the BMSR approach (Fig. 3c) revealed candidate biomarkers (Fig. 3d and Supplementary Fig. 20) that were distinctly separated from the bulk of non-contributing genes (i.e., those with coefficients near zero in both datasets). Candidate biomarkers included genes involved in cell proliferation [*IL7R*^15^ and *NIBAN2*^16^], cell cycle [*MAPK12*^17^], cell growth [*BCL2*^18^ and *S100A8*/*S100A9*^19^], apoptosis [*BCL2*^20^, *NIBAN2*^21^, *S100A8*/*S100A9*^22^], and drug response [*BCL2*^23^]). Notably, these genes were near the periphery of the ridge coefficient distribution (Fig. 3b), thus demonstrating the consistency of the two methods. As expected, candidate *GRD* biomarkers were also consistently correlated with response to *individual* drugs (Supplementary Fig. 21).

### Drug response is robustly predicted by gene expression

**Fig. 4:**
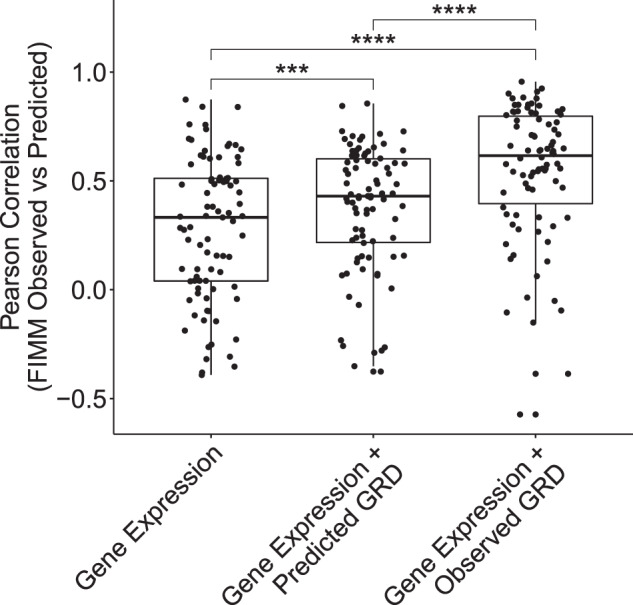
Expression-based predictions of drug response indicate concordance of independent ex vivo datasets and may be improved by incorporating general response across drugs. Performance (Pearson correlation between observed and model-predicted drug response; *y* axis) of ridge regression models trained on OHSU data and tested on FIMM data using genes as predictors (Gene Expression), genes and GRD predicted by applying ridge regression to gene expression (Gene Expression + Predicted GRD), or genes and GRD calculated from drug response data (Gene Expression + Observed GRD). Each point corresponds to a drug (*n* = 87). Drug *d* is excluded from observed and predicted GRD in modeling *d*’s response. ****One-sided paired Wilcoxon signed rank *p* < 0.0001; ***p < 0.001.

Responses could be significantly predicted (*p* < 0.01) using expression-based ridge regression for 31 of the 87 drugs (Fig. 4 and Supplementary Table 16; median *r* = 0.33; 25th–75th percentile = 0.04–0.51; all significant correlations were positive; see “Methods”). Significantly predicted drugs included the heat shock protein inhibitor tanespimycin, the immunomodulatory agent lenalidomide, the bromodomain inhibitor JQ1, two apoptotic modulators (nutlin-3 and venetoclax), and 26 kinase inhibitors. Prediction performance was correlated with the spread of response (i.e., IQR of unnormalized AUCs) in the FIMM validation dataset (Supplementary Fig. 22; *r* = 0.36; *p* = 6.19 × 10^−4^). These results reinforce the consistency of the two ex vivo studies demonstrated by the drug–drug correlations above.

Using predicted GRD as a model variable in addition to gene expression improved response-modeling performance (median *r* = 0.43; 25th–75th percentile = 0.17–0.60) relative to modeling based on gene expression variables alone (one-sided paired Wilcoxon signed rank *p* = 3.45 × 10^−4^; Fig. 4). Using observed, rather than predicted, GRD as a variable in addition to gene expression further improved performance (median *r* = 0.63; 25th–75th percentile = 0.39–0.75) relative to using gene expression alone (one-sided paired Wilcoxon signed rank *p* = 4.71 × 10^−11^; Fig. 4).

These results were largely independent of whether GRD was computed across common drugs or all drugs in a dataset (correlation *r* between common drug and all drug-based models of observed versus predicted drug response correlations ≥0.96; Supplementary Figs. 23 and 24). This was true despite decreased performance in predicting GRD across all drugs (*r* = 0.52; Supplementary Fig. 25) relative to across common drugs (*r* = 0.67; Fig. 3).

A previous study proposed a filtering strategy to combat experimental and technical noise anticipated in large-scale drug screens^24^. To ensure that our findings were robust to such noise, we developed and applied a related outlier-removal approach (Supplementary Figs. 26–31; see “Methods”). Since prediction performance was not significantly different after removing outliers (Supplementary Fig. 32; two-sided Wilcoxon rank-sum test *p* = 0.89; Supplementary Tables 17 and 18), subsequent analysis does not exclude outliers.

### Monocytic signature predicts venetoclax resistance

Having determined potential biomarkers of GRD above, we next asked whether responses of individual drugs were driven solely by GRD. To isolate drug-specific responses from general effects, we compared prediction performance of models using both gene expression and GRD as variables to that of models using only GRD (i.e., effectively drug–GRD correlations). Drugs showing the greatest specificity were the MEK1/2 inhibitors trametinib, PD184352, and selumetinib and the BCL-2 inhibitor venetoclax (Supplementary Fig. 33). Of these, gene-based prediction performance was highest for venetoclax (*r* = 0.84; *p* = 7.50 × 10^−8^; Supplementary Fig. 34A).

**Fig. 5:**
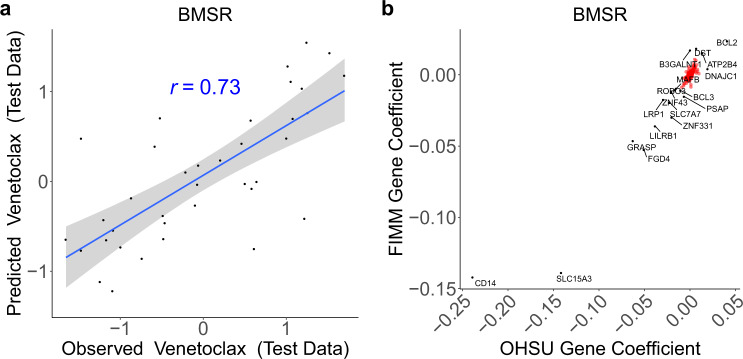
Integrative analysis reveals monocyte-associated biomarkers predictive of venetoclax resistance. **a** Observed (*x* axis) versus BMSR-predicted (*y* axis) venetoclax response. Expression-based Bayesian regression model trained using fivefold cross-validation on combined OHSU and FIMM datasets (*n* = 159) and tested on held-out fold yielding median performance across the fivefolds (*n* = 37). **b** Coefficients of genes (*n* = 2132) in OHSU (*x* axis) or FIMM (*y* axis) datasets following training of Bayesian regression modeling simultaneously on both datasets (*n* = 196). *r*: Pearson correlation; dashed line: identity line; blue line: linear regression fit; gray shading: 95% confidence interval. Labeled genes were those having extremal (Stouffer’s *p* < 0.01) combined coefficients across both datasets.

BMSR analysis revealed that venetoclax response had a positive association with the gene *BCL2* encoding the drug target, as well as strong negative correlations with *CD14* and *SLC15A3* (Fig. 5 and Supplementary Figs. 34 and 35). *SLC15A3* (Solute Carrier Family 15 Member 3) is highly expressed in monocytes at the protein^25^ and mRNA^26,27^ levels, while *CD14* encodes a canonical (classical) monocyte cell surface marker^28^. As such, BMSR analysis indicated that monocyte-associated genes are correlated with venetoclax resistance. We confirmed that genes having expression correlated with venetoclax resistance are enriched for a monocyte signature (*p* = 2.0 × 10^−4^; Supplementary Fig. 36 and Supplementary Table 19). Both results are consistent with findings from Kuusanmäki and colleagues that the myeloid differentiation stage of AML cells impacts venetoclax response, with monocytic cells exhibiting resistance to BCL-2 inhibition^9^.

As with GRD above, these candidate biomarkers contribute consistently (i.e., with the same sign) to the ridge regression model (Supplementary Fig. 34), but BMSR prioritizes a smaller set of features with large coefficients. As a comparison, we also applied minimum redundancy maximum relevance (mRMR), a method that performs feature selection within a single dataset to identify parsimonious feature sets^29,30^. mRMR identified a subset of the BMSR-prioritized, monocyte-associated genes across both datasets, but the individual genes selected differed. In particular, across 200 random (bootstrap) samples of the datasets, mRMR selected *SLC15A3* across all random samples in the OHSU dataset but only once in the FIMM dataset and, conversely, selected *PSAP* across all random samples in the FIMM dataset but only once in the OHSU dataset. mRMR identified all BMSR-prioritized, monocyte-associated genes across one or more random samples; however, no gene was selected more than twice across both datasets (Supplementary Table 20). Collectively, these results demonstrate the biological consistency of the BMSR, ridge regression, and mRMR analyses, while highlighting BMSR’s motivating contribution of identifying a small set of features concordantly across datasets.

Intriguingly, genes associated with venetoclax resistance were also enriched for T and/or B cell-mediated pathways (Supplementary Tables 21–25). Further, we observed that venetoclax response was positively associated with blast percentage of both peripheral blood (*r* = 0.37; two-sided Wilcoxon rank-sum test *p* = 4.6 × 10^−4^) and bone marrow (*r* = 0.43; *p* = 5.9 × 10^−5^; Supplementary Fig. 37). Nevertheless, several lines of evidence suggest that our results are unlikely to be compromised by impure leukemic samples and/or lymphocyte contamination. First, predicted levels of lymphocytes are lower and less variable than those of monocytes in both datasets (Supplementary Fig. 38). Second, genes associated with venetoclax resistance are more enriched for markers of monocytes and other cell types within the monocytic lineage (i.e., macrophages) than for markers of lymphocytes and are also strongly enriched in myeloid dendritic cells (Supplementary Table 19). Finally, we controlled for potential confounding effects by including lymphocyte levels as covariates in our ridge regression models. Our findings were consistent with the original ridge regression models: prediction results across drugs were highly correlated between the two models (*r* > 0.99; Supplementary Fig. 39) and genes contributing most to the lymphocyte-controlled model continued to show strong enrichments for markers of the monocytic lineage (Supplementary Table 26).

Because technical or biological variation may contribute noise to individual genes, we next sought to develop a robust signature of venetoclax resistance that would mitigate these fluctuations. To do so, we focused attention on monocyte-associated genes prioritized by BMSR (see “Methods”), which, in addition to *CD14* and *SLC15A3*, included *BCL3*, *LILRB1*, *LRP1*, *MAFB*, *PSAP*, and *SLC7A7* (Fig. 5b). We compressed the expression of these genes into a single enrichment-based signature of venetoclax resistance (see “Methods”).

**Fig. 6:**
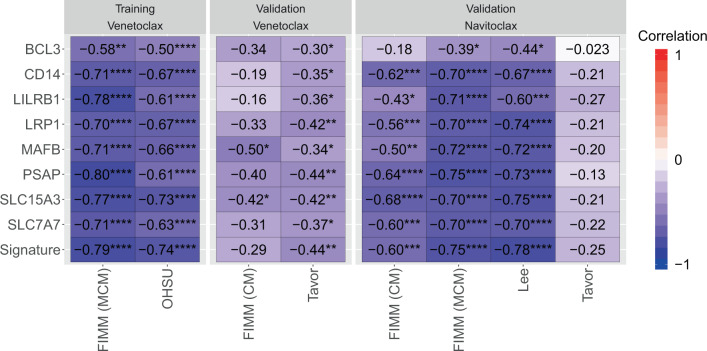
A monocyte expression signature robustly predicts resistance to BCL-2 inhibitors. Pearson correlation of response of the indicated drug (venetoclax or navitoclax; top) versus expression of the indicated gene or venetoclax monocyte signature (Signature) across FIMM (MCM), OHSU, FIMM (CM), Lee, or Tavor datasets. Dataset/drug combinations are indicated as “Training” if they were used to derive the signature and biomarkers and “Validation” otherwise. ****p < 0.0001; ***p < 0.001; **p < 0.01; *p < 0.05.

We sought to validate this signature (and its constituent genes) across several independent conditions, drugs, and/or ex vivo functional and transcriptomic profiles of AML (Fig. 6), including a study by Lee and colleagues of drug sensitivity that profiled the BCL-2/BCL-XL inhibitor navitoclax^31^, the ex vivo study by Tavor and colleagues that profiled both venetoclax and navitoclax^14^, and a second FIMM dataset that profiled venetoclax and navitoclax in a stroma-derived conditioned medium (CM) that differed from the mononuclear cell medium (MCM) of the above FIMM dataset^32^. The signature was inversely correlated with venetoclax response (i.e., correlated with resistance) in the Tavor dataset (*r* = −0.44; *p* = 3.2 × 10^−3^). It also trended with resistance in the FIMM CM dataset (*r* = −0.29; *p* = 0.17), with a lack of significance consistent with the specific dampening of venetoclax response in CM relative to MCM^32^. Despite this, the signature smoothed out the weak anti-correlations of several genes (e.g., *CD14* and *LILRB1*) as intended. The monocytic signature was strongly anti-correlated with navitoclax response in three datasets: FIMM (CM) (*r* = −0.60; *p* = 2.22 × 10^−4^), FIMM (MCM) (*r* = −0.75; *p* = 1.06 × 10^−7^), and Lee (*r* = −0.78; *p* = 2.83 × 10^−7^). All genes in the signature, with the exception of *BCL3*, also validated against navitoclax in these three datasets, though their correlation was often not as strong as that of the signature itself. The signature trended with navitoclax resistance in the Tavor dataset (*r* = −0.25; *p* = 0.11), with *BCL3* again having the weakest association.

As a further validation of the robustness of the signature and of the generality of our Bayesian approach, we used BMSR to jointly analyze the Tavor and FIMM (CM) datasets. Following the approach above, we defined a signature from the monocyte-associated genes prioritized by the analysis. This revised signature was highly correlated with the original FIMM/OHSU-derived signature in the FIMM, FIMM (CM), OHSU, and Tavor datasets (*r* ≥ 0.75; Supplementary Fig. 40). Finally, to demonstrate its applicability to more than two datasets, we similarly applied BMSR to three datasets, FIMM, OHSU, and Tavor, and again observed that the resulting signature was highly correlated with the original FIMM/OHSU-derived signature (*r* ≥ 0.95; Supplementary Fig. 41).

Several biomarkers of venetoclax response in AML have been proposed at the protein [BCL-XL, MCL-1, and BCL-2^33^] and mRNA [*BCL2*, *BCL2*/*MCL1* ratio, *BCL2A1*, *CD11b*, *CD14*, *CD68*, *CD86*, *CLEC7A* (*CD369*), *HOX* gene family members, *MCL1*, *S100A8*, and *S100A9*^9,34–37^] levels. Of these, the monocytic signature, *BCL2A1*, *CD68*, *CD86*, and *CLEC7A* were robust predictors across both BCL-2-inhibitors (venetoclax or navitoclax), different cell culture conditions, and datasets, with none performing best across all conditions (Supplementary Fig. 42). Strikingly, all five were strongly correlated with one another (Supplementary Fig. 43) and are highly expressed in monocytes^27,38^. These findings validate our own monocytic signature and previously proposed, monocyte-associated biomarkers as predictors of resistance to BCL-2 inhibition in AML.

### Monocytic signature predicts MEK inhibitor response

Similar to venetoclax, gene expression data improved the prediction performance of the three MEK inhibitors, trametinib, PD184352, and selumetinib, beyond that provided by GRD alone (Supplementary Fig. 33). We therefore next sought to determine biomarkers of their response. As the response of the MEK inhibitors are strongly correlated with one another (*r* > 0.48; Supplementary Fig. 44), we developed a Bayesian multi-source multi-task regression (BMSMTR) approach that simultaneously analyzes multiple datasets (i.e., multi-source, as above) and also multiple drugs (i.e., multi-task; see “Methods”). Joint analysis of trametinib, PD184352, and selumetinib with BMSMTR identified monocytic genes as candidate biomarkers, including *LRP1* and *CD300E* (Supplementary Fig. 45), that are *positively* associated with MEK inhibitor response. Hence, we hypothesized that the monocytic signature defined above to be correlated with venetoclax *resistance* would be correlated with MEK inhibitor *response*. Indeed, we observed a positive correlation between the response of each of the three MEK inhibitors and the monocytic signature in both the OHSU and FIMM datasets, where BCL-2 inhibitor response is inversely correlated with MEK inhibitor response (Supplementary Fig. 44). The association did not hold in the FIMM CM dataset, where BCL-2 inhibitor response is not significantly (inversely) (Supplementary Fig. 44) correlated with MEK inhibitor response and where our hypothesis would not be expected to hold.

## Discussion

We demonstrated consistency between two large-scale AML studies that profiled ex vivo drug sensitivity and gene expression. In both datasets, we observed that patient-derived samples exhibited a GRD, i.e., a sample often responded uniformly across drugs independent of target or mechanism of action—relatively strongly to all drugs or relatively weakly to all drugs. GRD was further associated with clinical endpoints. Finally, we developed a BMSR method for biomarker discovery and applied it to reveal a robust monocytic signature of BCL-2 inhibition in AML.

We demonstrated that the two studies were consistent: first, drug–drug correlations were conserved across studies (Supplementary Fig. 3); second, the mean response of each drug across patients relative to that of the other drugs was also conserved across studies (Supplementary Fig. 4); and, finally, regression models trained on gene expression data in one study predicted response in the second, independent study for 31 of the 87 drugs (Fig. 4). Similar efforts comparing^39^ in vitro drug screens^40,41^ reported discordance in drug responses across the datasets. This finding spurred considerable activity in the research community^24,42–47^, which ultimately resolved certain discrepancies between the Cancer Cell Line Encyclopedia (CCLE) and the Genomics of Drug Sensitivity in Cancer (GDSC) studies, in part, by harmonizing curve fitting methods applied across datasets and by quantifying drug response via a modified AUC^44,48^. We also employed AUC since it both intuitively and empirically (Supplementary Figs. 1 and 2) provides a more robust summary of a multi-parameter curve fit than does a single parameter such as EC_50_ or IC_50_. A retrospective re-analysis of these in vitro studies applied quality control filters to exclude curves that grossly violated the assumptions that sensitivities range between 0 and 100% and that they increase monotonically with drug concentration^24^. We developed and applied related quality control measures (Supplementary Figs. 26–31) and found they had little impact on prediction performance of most drugs (Supplementary Fig. 32). Nevertheless, even low-frequency technical noise is expected to be observed in large-scale studies, and hence, we concur with Safikhani and colleagues^24^ that its impact should be carefully investigated, as we have done here.

Geeleher and colleagues^13^ reported a phenomenon similar to GRD—GLDS—across cell lines spanning cancer types^40,41,49,50^. They showed that conditioning on GLDS eliminated spurious biomarker predictions while identifying evidence-supported biomarkers that otherwise went undetected. We similarly showed that including GRD in prediction models improved overall accuracy (Fig. 4), thus demonstrating that the general trend detected in vitro is active in patient-derived AML samples as well. Thus, precision oncology studies should account for both target-specific and target-agnostic effects when correlating drug response with biomarkers.

Our findings directly relate this trend to clinical endpoints: increased GRD is associated with complete response to induction therapy and to improved overall survival (Fig. 2). This generalizes the previous observation that ex vivo response to individual drugs may be correlated with AML remission status^31^. Further, we found that patients with *FLT3*-ITD mutations have higher GRD. This result held even when GRD was calculated from subsets of drugs that excluded *FLT3* inhibitors. Relatedly, Tavor and colleagues found that patient-derived samples with high sensitivity across TKIs and several other drugs were enriched in *FLT3*-ITD mutations relative to resistant samples^14^. Collectively, these results suggest that *FLT3*-ITD mutations may confer a generalized drug sensitivity. One such mechanism for doing so may be through its association with reduced levels of the ABCB1 drug efflux pump^51^. Indeed, our expression-based analysis revealed that *ABCB1* expression was inversely correlated with GRD. Nevertheless, prior results relating *ABCB1* expression to ex vivo response to agents used in induction therapy have been inconsistent^51–53^. Additionally, the modest correlation of candidate biomarkers with GRD (Supplementary Fig. 20), particularly relative to the consistent correlation observed for venetoclax biomarkers (Supplementary Fig. 35), suggests that mechanisms underlying GRD may involve a complex interplay of multiple genes, possibly conditioned on *FLT3* status.

We developed BMSR to mitigate factors that limit reproducibility in prioritizing biomarkers in high-dimensional gene feature spaces, including small sample sizes, correlated expression of functionally related genes, technical variation across datasets, and heterogeneity of patient populations^54–57^. It does so by performing integrated analysis^11,58^ across multiple heterogeneous datasets to increase cumulative sample size, both of which reduce the likelihood of overfitting. BMSR shares information across the datasets: gene expression values (or, more formally, the gene expression coefficients) in each dataset are modeled as arising from a shared prior distribution. BMSR differs from meta-analysis methods that first analyze datasets independently before combining effect sizes or *p* values across datasets. By regularizing the prior’s hyperparameters, BMSR effectively selects features simultaneously across the two datasets to prioritize a sparse set of biomarkers. This is manifested in a small number of genes having coefficients that are well separated from the majority of gene coefficients in both datasets, in contrast to independent ridge regression analysis across the two datasets in which coefficients are evenly distributed with no clear separation indicating candidate biomarkers (Fig. 3 and Supplementary Fig. 34).

We demonstrated BMSR’s generalizability by jointly analyzing different pairs of datasets [FIMM and OHSU; FIMM (CM) and Tavor] and by analyzing a dataset trio (FIMM, OHSU, and Tavor; Supplementary Figs. 40 and 41). As such, we anticipate BMSR will have broad applicability in integrative biomarker studies. However, such analyses are often plagued by the effort required to harmonize multiple datasets. To address this issue, we collaborated with the developers of ORCESTRA in the Haibe-Kains laboratory to make several of the datasets (the Tavor and OHSU datasets) more broadly and easily accessible^59^. ORCESTRA is a cloud-based platform that provides automated processing of pharmacogenomic profiles and packages them into a fully documented and DOI-indexed “PharmacoSet” (PSet). These PSets are compatible with PharmacoGx, an open-source computational framework that facilitates integrative studies of multiple pharmacogenomic datasets through routines for standardized access and analysis^60^. To provide a template for others in applying BMSR for integrative analysis, we included a demo in our BMSR GitHub repository (https://github.com/suleimank/bmsr) that downloads the Tavor (10.5281/zenodo.4585705) and OHSU (10.5281/zenodo.4582786) datasets from ORCESTRA and uses the functions of PharmacoGx to predict biomarkers of venetoclax response. Through our ongoing collaboration, we will also make these datasets available through PharmacoDB (https://pharmacodb.ca), a web application that similarly assembles pharmacogenomic datasets into a single database for cross-study analyses^61^. The online, interactive capabilities of PharmacoDB will make these resources accessible beyond computational biologists and those with programming skills.

BMSR revealed that venetoclax resistance is correlated with expression of monocyte-associated genes (Fig. 5). We combined these genes into a single signature that is robust to the variations of its individual genes^62^ across datasets (Fig. 6). Additionally, we showed that the venetoclax-derived signature strongly predicts resistance to the BCL-2/BCL-XL inhibitor navitoclax. Resistance to both venetoclax and navitoclax was significantly correlated with the signature in standard culture conditions (MCM), though the trends in a stroma-derived CM reached significance only for navitoclax and not for venetoclax. This may have resulted from the reduced sensitivity of AML cells to venetoclax observed in CM relative to MCM, mediated by a switch from BCL-2- to BCL-XL-dependent cell survival that has a less pronounced effect on navitoclax^32^.

Our findings extend and support recent reports demonstrating that monocytic cells are resistant to BCL-2 inhibition, whereas myeloid progenitors exhibit sensitivity. Kuusanmäki and colleagues showed that differentiated cells from AML samples expressing monocytic markers are less sensitive to venetoclax than immature blasts^9^. They further showed that sensitivity to venetoclax decreased along the differentiation spectrum, from less differentiated AML samples [French-American-British subtype M1] to more differentiated samples with significant monocytic differentiation (M5 subtype). They concluded by associating increased expression of monocyte markers *CD14*, *CD11b*, *CD86*, and *CD68* with decreased ex vivo responsiveness to venetoclax. Among other identified biomarkers, Zhang and colleagues also implicated high expression of *CD14*, as well as the monocyte-associated *CLEC7A* (*CD369*), with reduced sensitivity to venetoclax^36^. We provide independent validation of these biomarkers, confirming that leukemic cells expressing high levels of *CD68*, *CD86*, and *CLEC7A*, in particular, are more resistant to BCL-2 inhibition via both venetoclax and navitoclax (Supplementary Fig. 42). Further, we identified a mostly non-overlapping, but strongly correlated (Supplementary Fig. 43), set of genes highly expressed in monocytes: *BCL3*, *CD14*, *LILRB1*, *LRP1*, *MAFB*, *PSAP*, *SLC15A3*, and *SLC7A7*. We combined these genes into a per-sample monocytic score using a straightforward approach that did not require additional training or parameter turning. This signature was competitive with *CD68*, *CD86*, and *CLEC7A* across BCL-2 inhibitors, culture conditions, and datasets. Since neither the signature nor the genes nominated by these two studies outperformed the others across all conditions, it may be beneficial to combine them for increased robustness, much as the signature itself smoothed out variation in its constituent genes. Such an approach should be validated in the future using independent data.

These results are likely to be of clinical relevance, as Pei and colleagues demonstrated that phenotypically primitive AML is sensitive to venetoclax in vivo, whereas monocytic AML is more resistant^10^. Further, they showed that venetoclax (in combination with azacitidine) selects for monocytic subclones present at diagnosis. This resistance is associated with a loss of BCL-2 expression and a concomitant shift to MCL1 for survival that is inherent in monocytic differentiation. MCL1 is itself stabilized by extracellular signal-regulated kinase (ERK)^63,64^. As such, targeting ERK via a MEK inhibitor has been shown to sensitize cells to the BCL-2/BCL-XL inhibitor ABT-737 in vitro and in xenograft models^65^. Targeting MCL1 has also been shown to forestall acquired resistance to venetoclax in vitro^66^, while leukemic blasts resistant to venetoclax are sensitive to MEK inhibition via trametinib ex vivo^9^. Finally, combination of venetoclax with the MEK inhibitor cobimetinib exhibited synergy in vitro, inhibited growth ex vivo, and reduced leukemia burden in xenografts^12^. Our work has implications for the rational selection of patient groups for combination therapies targeting these two pathways. Since the monocytic signature is correlated with BCL-2 inhibitor resistance and MEK inhibitor response, it may provide a means of prospectively identifying patients for combination treatment.

## Methods

### Drug–response curve fitting and filtering

We fit 3- (LL3) and 4-parameter log-logistic (LL4) curves to the dose–response data using PharmacoGx^60^ and drc^67^ in R^68^, respectively. We excluded non-AML patients or those exhibiting gross dissimilarities across replicates from analysis. We excluded any drug–sample pair having a concentration range outside the most common (dataset-specific) concentration range for that corresponding drug. We further excluded a drug–sample pair if it did not include all concentration points and only analyzed one sample per drug–patient pair (see Supplementary Methods). Additionally, we assessed the impact of an outlier-removal strategy that excluded drug–sample pairs: (1) whose fits were not monotonically increasing, (2) that had large differences between fits that did (LL3) and did not (LL4) constrain the curve to asymptote to zero response at low drug concentration, or (3) had a replicate screen (technical in OHSU and biological in FIMM) to which it strongly differed (Supplementary Figs. 26–31). However, we found that this outlier-removal strategy had little impact on prediction performance (Supplementary Fig. 32) and hence did not apply it in the analyses.

### Bayesian regression analysis

BMSR models the gene coefficients in each dataset as a sample from a shared, underlying data generating distribution and by regularizing the mean of that distribution to provide parsimony. Formally, it performs joint regression across all datasets constrained to a set of *N*_G_ common genes as

1 $$\begin{array}{l}{{\bf{y}}}^{(d)} \sim N({X}^{(d)}{{\boldsymbol{\beta }}}^{(d)},{\sigma }^{(d)}{\bf{I}})\\ {{\boldsymbol{\beta }}}^{(d)} \sim N({\boldsymbol{\beta }},0.5)\\ {\beta }_{{\rm{g}}} \sim N(0,{\lambda }_{{\rm{g}}}^{2}{\tau }^{2})\ ,\end{array}$$

where ${{\bf{y}}}^{(d)}\in {{\mathbb{R}}}^{{N}_{d}\times 1}$ is the response vector for a particular drug across the *N*_*d*_ patient samples in dataset *d* ∈ {FIMM, OHSU}, ${X}^{(d)}\in {{\mathbb{R}}}^{{N}_{d}\times {N}_{{\rm{G}}}}$ is the corresponding expression matrix over *N*_G_ genes, ${{\boldsymbol{\beta }}}^{(d)}\in {{\mathbb{R}}}^{{N}_{{\rm{G}}}\times 1}$ is the gene regression coefficient vector, **I** is the *N*_G_ × *N*_G_ identity matrix, and the standard deviation *σ*^(*d*)^ has a non-informative noise prior

$${\sigma }^{(d)} \sim {\rm{IG}}(1,1)\ ,$$

with IG(*α*, *β*) the Inverse Gamma distribution.

BMSR models coefficient vectors ***β***^(*d*)^ using the joint hierarchical prior with the shared mean coefficient vector ${\boldsymbol{\beta }}\in {{\mathbb{R}}}^{{N}_{{\rm{G}}}\times 1}$. It regularizes the scalar components *β*_g_ using the Finnish horse-shoe prior^69^

$$\begin{array}{l}{\lambda }_{{\rm{g}}} \sim {C}^{+}(0,1)\\ \tau \sim {C}^{+}(0,{\tau }_{0})\\ {\tau }_{0}=\frac{{p}_{0}}{{N}_{{\rm{G}}}-{p}_{0}}\frac{{\sum }_{d}{\sigma }^{(d)}}{\sqrt{{N}_{d}}}\ ,\end{array}$$

where *C*^+^(*μ*, *σ*) is the half-Cauchy distribution with location *μ* and scale *σ*, the scalar *λ*_g_ induces localized gene-wise regularization, and the scalar *τ* is the global regularization parameter that induces the number of active genes (*p*_0_) a priori. Collectively, this formulation encourages the dataset-specific coefficients to either have large magnitude in both datasets (i.e., representing genes whose expression makes a large contribution to the response) or small magnitude in both datasets (i.e., genes with little or no contribution) and to have the same direction (i.e., sign) in both datasets.

BMSMTR simultaneously analyzes multiple datasets (i.e., multi-source, as in BMSR) and also multiple drugs (i.e., multi-task) in a set of drugs ${\mathcal{I}}$. It does so by generalizing Eq. (1) according to

$${{\bf{y}}}^{(d,i)} \sim N\left({X}^{(d)}{{\boldsymbol{\beta }}}^{(d)}{w}^{(i)},{\sigma }^{(d)}{\bf{I}}\right)\ ,$$

with the response vector ${{\bf{y}}}^{(d,i)}\in {{\mathbb{R}}}^{{N}_{d}\times 1}$ for dataset *d* and drug $i\in {\mathcal{I}}$ distributed about a mean that is a product of a factor *X*^(*d*)^***β***^(*d*)^ common to drugs in ${\mathcal{I}}$ and a factor *w*^(*i*)^ ~ *N*(0.5, 0.5) specific to drug *i*.

The BMSR and BMSMTR models were implemented in STAN^70^, a platform for statistical modeling that provides efficient automatic procedures for Bayesian statistical inference. We performed inference using MCMC sampling with 500 samples of the posterior and a burn-in of 500. BMSR and BMSMTR are available at https://github.com/suleimank/bmsr.

### Statistical analysis

Statistical analyses were performed in R^68^. Wilcoxon rank-sum test was performed using wilcox.test. Fisher’s exact test was performed with fisher.test. Linear regression was performed with lm. Forest plot for multivariate linear regression was generated using the forestmodel package.

### Gene expression analysis

Ridge regression was performed using glmnet^71^. Genes used as variables in (ridge, BMSR, and BMSMTR) regression models were filtered to exclude those with low expression (Supplementary Figs. 14–17) and low variance (Supplementary Figs. 18–19). Gene set enrichment was performed using fgsea^72^, including relative to the set of monocyte marker genes defined in CIBERSORT^73^. Genes differentially expressed in monocytes relative to other cell populations were determined by applying limma^74^ to the expression dataset GSE24759^26^. Immune cell fractions were computed with CIBERSORT using non-log expression data and parameters QN=TRUE, absolute=TRUE, and abs_method=“no.sumto1”. Expression of the genes *BCL3*, *CD14*, *LILRB1*, *LRP1*, *MAFB*, *PSAP*, *SLC15A3*, and *SLC7A7* was compressed into a single enrichment score using GSVA^75^. This enrichment score is the monocytic signature. Additional details are provided in Supplementary Methods.

### Reporting summary

Further information on research design is available in the Nature Research Reporting Summary linked to this article.

## Supplementary information

Supplementary Information

Supplementary Data 1

Reporting Summary

## Data Availability

We re-analyzed four datasets in this study. The OHSU/Beat AML dataset is available on the Synapse data-sharing platform (https://www.synapse.org/#!Synapse:syn2942337/wiki/390658), at dbGaP (study ID 30641 and accession ID phs001657.v1.p1), as supplementary data within the original manuscript^4^, and via ORCESTRA (10.5281/zenodo.4582786). Additionally, the OHSU dataset can be interactively browsed using Vizome (www.vizome.org). Our re-analysis of the OHSU/Beat AML drug response data are included in Supplementary Tables 1 and 17. The FIMM (MCM) and FIMM (CM) RNA-seq data are available for re-analysis upon request at a secure and GDPR-compliant data analysis data lake environment. Only aggregate data can be downloaded from the data lake. The Lee dataset is available as Supplementary Data within the original manuscript^31^. The Tavor dataset is available via ORCESTRA (10.5281/zenodo.4585705)^14^.

## Code availability

The BMSR and BMSMTR code is available on GitHub (https://github.com/suleimank/bmsr).
