# Supplementary material for: Bayesian multi-source regression and monocyte-associated gene expression predict BCL-2 inhibitor resistance in acute myeloid leukemia
Source: NPJ Precis Oncol. 2021 Jul 23;5:71. doi: 10.1038/s41698-021-00209-9 (PMC8302655; doi:10.1038/s41698-021-00209-9)
Supplement: Supplementary file 1 — Supplementary Information [file 41698_2021_209_MOESM1_ESM.pdf]

Bayesian multi-source regression and monocyte-associated gene expression predict BCL-2 inhibitor resistance in acute myeloid leukemia

Brian S. White[*,†,‡,1], Suleiman A. Khan[†,2], Mike J. Mason[1], Muhammad Ammad-ud-din[2], Swapnil Potdar[2], Disha Malani[2], Heikki Kuusanmäki[2,3], Brian J. Druker[4,5], Caroline Heckman[2], Olli Kallioniemi[2,6], Stephen E. Kurtz[5], Kimmo Porkka[7], Cristina E. Tognon[4,5], Jeffrey W. Tyner[5], Tero Aittokallio[§,2,8,9,10], Krister Wennerberg[§,2,3], and Justin Guinney[§,1,11]

[1]Computational Oncology, Sage Bionetworks, Seattle, USA
[2]Institute for Molecular Medicine Finland (FIMM), Helsinki Institute of Life Science (HiLIFE), University of Helsinki, Helsinki, Finland
[3]Biotech Research & Innovation Centre (BRIC) and Novo Nordisk Foundation Center for Stem Cell Biology (DanStem), University of Copenhagen, Copenhagen, Denmark
[4]Howard Hughes Medical Institute, Portland, USA
[5]Division of Hematology and Medical Oncology, Knight Cancer Institute, Oregon Health & Science University, Portland, USA
[6]Scilifelab, Karolinska Institute, Solna, Sweden
[7]HUS Comprehensive Cancer Center, Hematology Research Unit Helsinki and iCAN Digital Precision Cancer Center Medicine Flagship, University of Helsinki, Finland
[8]Department of Mathematics and Statistics, University of Turku, Turku, Finland
[9]Department of Cancer Genetics, Institute for Cancer Research, Oslo University Hospital, Oslo, Norway
[10]Centre for Biostatistics and Epidemiology (OCBE), University of Oslo, Norway
[11]Department of Biomedical Informatics and Medical Education, University of Washington, Seattle, USA

July 14, 2021

Supplementary Methods

Drug curation

OHSU and FIMM drug identifiers were reconciled using the Drug Target Commons [1].

Fitting drug response curves

Prior to curve fitting, raw drug response data were harmonized so that concentrations were expressed in nM (OHSU concentrations were converted from M) and responses were expressed both as percent viability (FIMM responses were converted from percent inhibition by subtracting them from 100%) and as percent inhibition (OHSU responses were converted from percent viability by subtracting them from 100%).

Drug inhibition values y_x at concentration x (in linear, nM space) were fit to the 4-parameter log-logistic function ($LL4$)

$$LL4(x) = c + \frac{d - c}{1 + e^{b[\log(x) - \log(e)]}} \tag{S1}$$

and the 4-parameter logistic function ($L4$)

$$L4(x) = c + \frac{d - c}{1 + e^{b(x-e)}} \tag{S2}$$

where e is the EC_{50} (in linear space) and b is the slope parameter, independently for each drug-sample replicate. These functions do not constrain the asymptotes c and d (Supplementary Fig. 26A). Fits were calculated using the function `drm` in the R package `drc` [2]. Conceptually, inhibition values y_x at concentration x were also fit to the 3-parameter log-logistic function ($LL3$)

$$LL3(x) = \frac{d}{1 + e^{b[\log(x) - log(e)]}} \tag{S3}$$

again independently for each drug-sample replicate (Supplementary Fig. 26A). This form effectively constrains one of the asymptotes to zero sensitivity, while the second (d) is left free. Whether zero is the low- or high-concentration asymptote is controlled by the sign of the slope parameter b. However, forcing zero

*Corresponding author: bstephenwhite@gmail.com
†Contributed equally
‡Current affiliation: The Jackson Laboratory for Genomic Medicine, Farmington, CT, USA
§Contributed equally

to be the low-concentration sensitivity asymptote (by constraining the sign of b) would negatively affect the goodness of fit for curves that are essentially flat [i.e., those with (positive or negative) slope b near zero]. Instead, we evaluate the impact of filtering sensitivity curves that are non-monotonically increasing (see section Filtering drug response curves and calculating AUCs below). Here, e retains its interpretation from Eq. S1 as the EC_{50}. In practice, the viability values $\widehat{y_x} \equiv 100 - y_x$ are fit to x using the `logLogisticRegression` function in the R package `PharmacoGx` [3] (with parameter `trunc=FALSE` to prevent viability values from being truncated to lie between 0% and 100% prior to curve fitting), which is equivalent to fitting the *sensitivity* values y_x to Eq. S3.

Drug response curve quality of fit metrics

To ameliorate the noise previously observed in large-scale drug screens [4], we independently filtered the FIMM and OHSU *ex vivo* functional data. Briefly, we fit 3- ($LL3$) and 4-parameter log-logistic ($LL4$) curves to the dose-response data. We excluded non-AML patients or those exhibiting gross dissimilarities across replicates from analysis. We excluded any drug-sample pair having a concentration range outside the most common (dataset-specific) concentration range for that corresponding drug. We further excluded a drug-sample screen if it did not include all concentration points and only analyzed one sample per drug-patient pair. Additionally, we assessed the impact of an outlier-removal strategy that excluded drug-sample pairs: (1) whose fits were not monotonically increasing; (2) that had large differences between fits that did ($LL3$) and did not ($LL4$) constrain the curve to asymptote to zero response at low drug concentration; or (3) had a replicate screen (technical in OHSU and biological in FIMM) to which it strongly differed (Supplementary Figs. 26-31). However, we found that this outlier-removal strategy had little impact on prediction performance and, hence, did not apply it elsewhere. We summarized drug response as area under the dose-response curve (AUC), which was more stable across $LL3$, $LL4$, and 4-parameter logistic ($L4$) curve fits than EC_{50} (Supplementary Figs. 1-2).

Four quality of fit metrics were defined for each drug-sample pair and subsequently used for filtering and outlier removal:

1. $LL4(x_{\min}) - LL4(x_{\max})$: the difference between the $LL4$-predicted sensitivity at the minimum ($x_{\min}$) and maximum ($x_{\max}$) concentrations (Supplementary Fig. 26B). $LL4(x)$ was evaluated at x by plugging the parameters returned from `drm` into Eq. S1.

2. $\delta_{\max}$: the maximum change in sensitivity, $\delta_{\max} = \max_i \text{sensitivity}(x_{i-1}) - \text{sensitivity}(x_i)$, between neighboring concentration points x_{i-1} and x_i (Supplementary Fig. 26C).

3. RMSE ($LL4$): Root-mean-square error, RMSE $= \sqrt{\sum_i \delta_i}$, calculated from residuals $\delta_i = |LL4(x_i) - \text{sensitivity}(x_i)|$ between the $LL4$-predicted and observed drug sensitivities (Supplementary Fig. 26D). The residuals

δ_i were calculated by invoking the function `residuals` from package `drc` on the fit object returned from `drm`.

4. $\|LL3 - LL4\|_{L1}$: integral of absolute difference (i.e., the L^1-norm) in $\log_{10}$ space between $LL3$ and $LL4$ fits, $\|LL3 - LL4\|_{L1} = \int_{x_{\min}}^{x_{\max}} |LL3(x) - LL4(x)| d\log_{10}(x)$ (Supplementary Fig. 26E). The integral was calculated numerically by passing the integrand $\frac{1}{x\log(10)}|LL3(x) - LL4(x)|$ to the R function `integrate` and evaluating from $x_{\min}$ to $x_{\max}$ (in linear space). $\frac{1}{x\log(10)}$ is the Jacobian resulting from the transformation $d\log_{10}(x) = \frac{dx}{x\log(10)}$ needed to perform the integral in linear space. As above, $LL4(x)$ was evaluated at x by plugging the parameters returned from `drm` into Eq. S1. $LL3(x)$ was evaluated at x (in linear space) by converting the predicted fractional viabilities returned by the function `.Hill` in package `PharmacoGx` into predicted percent sensitivities by subtracting them from one and multiplying by 100. Fractional viabilities were calculated as `.Hill(log10(x), pars=c(HS, Einf/100, log10(EC50)))` where `HS`, `Einf`, and `EC50` are the fit parameters returned by `logLogisticRegression`.

Filtering drug response curves and calculating AUCs

The following steps were applied to filter noise and remove outliers in the OHSU and FIMM (MCM) datasets, but not the FIMM (CM) dataset. The steps were applied in the order indicated, though some steps were used only to remove outliers. We found our analysis to be robust to outliers and hence outliers were removed only in assessing their impact (Supplementary Fig. 32). The table below indicates whether the step was included in routine and/or outlier removal analysis. It also indicates the corresponding column in Supplementary Tables 1 and 17.

1. Restrict to AML samples. i.e., in OHSU require `dxAtSpecimenAcquisition == "ACUTE MYELOID LEUKAEMIA (AML) AND RELATED PRECURSOR NEOPLASMS"` and in FIMM (MCM) require `diagnosis == "C92.0 Acute myeloid leukaemia [AML]"`.

2. In the published OHSU dataset, drug sensitivities above 100% have been truncated to 100%. Multiple truncated sensitivities lead to ambiguity in defining the response and/or in assessing outliers. Hence, we remove any curve having multiple truncated sensitivities. We do allow at most one truncated sensitivity, anticipating that it will be filtered by subsequent steps if it is inconsistent with the remaining non-truncated sensitivities (e.g., is non-monotonic with respect to them).

3. Exclude curves that do not have the expected (i.e., most common) number of concentration points in their respective dataset: seven concentration points in the OHSU dataset and five concentration points in the FIMM dataset. Exceptions with too many concentration points may reflect replicates that are not individually labeled as such, whereas exceptions with

too few points will not capture the concentration-response relationship as well as those with the expected number of points.

4. Exclude patients with dissimilar replicates, i.e., exclude patients having two samples i and j or two replicates i and j of the same sample whose mean absolute difference in response AUC across all drugs $\mathcal{D}$, exceeds 10: $\frac{1}{|\mathcal{D}|}\sum_{d\in\mathcal{D}}|AUC_{i,d} - AUC_{j,d}| > 10$. This excludes the 12 patients 652, 1153, 1353, 1730, 1989, 2001, 2254, 2314, 2443, 2685, 2694, and 4201 from the OHSU dataset and the two patients FHRB_784 and FHRB_1064 from the FIMM dataset.

5. Exclude any curve for drug d having a concentration range that does not encompass the most common concentration for d, as defined individually for each dataset. We (re-)evaluated the drug response AUCs for each drug d using its respective common concentration range as the integration bounds. Note that we do not enforce the same concentration range for a given drug across datasets.

6. Filter curves having large δ_{max} or RMSE ($LL4$). We assume that δ_{max} and RMSE ($LL4$) reflect technical noise in the dose-response data and that this noise is random and independent across drug-sample screens, including across replicates. Hence, whereas the responses across replicates (biological or technical) would otherwise be expected to be similar [as measured by the root-mean-square distance (RMSD) between responses of the replicates], noise reflected in δ_{max} and RMSE ($LL4$) will reduce their similarity (i.e., increase the RMSD). Our general strategy is to maximize the difference between (the distribution of) RMSDs of replicate pairs that do and do not pass the δ_{max} and RMSE ($LL4$) filters. To simplify, we focus on pairwise RMSDs by using the first two replicates (if more than two). For given thresholds in δ_{max} and RMSE ($LL4$), we separate replicates into those in which both drug-sample screens pass the threshold and those in which one or both do not. We then use a one-sided Wilcoxon test of the difference between the RMSDs calculated from replicates that pass the threshold and those that do not. We search the space of thresholds in the range 2 to 100 in steps of 1 for δ_{max} and in the range 2 to 40 in steps of 0.5 for RMSE ($LL4$). We choose the thresholds that minimize the Wilcoxon p-value. Note that minimizing intra-replicate RMSDs using $\|LL3 - LL4\|_{L1}$ would not be appropriate: unlike δ_{max} and RMSE ($LL4$), large $\|LL3 - LL4\|_{L1}$ (generally indicative of noisy data) is not independent across screens (within the pair). For example, if a patient is not sensitive to a given drug, replicates derived from that patient will exhibit low, near-zero sensitivities for that drug, which may be negative due to technical variance. This would lead to large $\|LL3 - LL4\|_{L1}$ values, indicating, as intended, differences in the $LL3$ fit (that will not model negative sensitivities) and the $LL4$ fit (that does) and, hence, that the dose-response data can not be robustly fit and should be excluded. Nevertheless, the RMSD between these replicates within the pair could be small,

in contrast to the assumption that large values in the fit metrics [$\delta_{\max}$, RMSE ($LL4$), or $\|LL3 - LL4\|_{L1}$] should correlate with large RMSD. The $\delta_{\max}$ cutoff is $\delta^*_{\max} = 24$ in the OHSU dataset and $\delta^*_{\max} = 21$ in the FIMM dataset (Supplementary Figs. 27 and 28). The RMSE ($LL4$) cutoff is RMSE $(LL4)^* = 7$ in the OHSU dataset and RMSE $(LL4)^* = 11.5$ in the FIMM dataset.

7. Filter curves having large $\|LL3 - LL4\|_{L1}$. $LL3$ fits constrain the modeled sensitivity to be non-negative and further fix the lower asymptote at zero. $LL4$ fits impose no such constraint. As such, we hypothesize that dose-response data with sensitivities below 0% will be artificially constrained by the $LL3$ model, but not by the $LL4$ model, and hence the difference between the two fits, $\|LL3 - LL4\|_{L1}$, will be large. As such, we define a null distribution of $\|LL3 - LL4\|_{L1}$ values over a subset of data likely enriched for non-noisy fits (i.e., those passing the above filters and having sensitivities within the expected range of 0% to 100%) and exclude dose-response data (including those outside this expected range) having $\|LL3 - LL4\|_{L1}$ values that are outliers with respect to this null distribution. Specifically, we use a gamma distribution as the null distribution (by using the method of moments to define the mean μ and variance σ^2 of the "non-noisy fits" and translating them to the gamma scale and shape parameters as σ^2/μ and μ^2/σ^2, respectively) and filter any of the n dose-response curves passing the above six filters with a $\|LL3 - LL4\|_{L1}$ value greater than would be expected by chance (i.e., the smallest $\|LL3 - LL4\|_{L1}$ value at which the cumulative distribution function reaches $1/n$). The $\|LL3 - LL4\|_{L1}$ cutoff $\|LL3 - LL4\|^*_{L1} = 8.7$ in the OHSU dataset (where $n = 21,128$) and $\|LL3 - LL4\|^*_{L1} = 6.7$ in the FIMM dataset (where $n = 11,511$; Supplementary Figs. 29 and 30). The cumulative distribution function is inverted using the `qgamma` function in R.

8. Filter curves having large $LL4(x_{\min}) - LL4(x_{\max})$, which should nominally be in the range -100% to 0%. We hypothesize that dose-response curves outside of this range will be enriched for noisy data that should be filtered. Similar to step 7 above, we define a null distribution over a subset of the data expected to be enriched for non-noisy drug responses (namely, that passing the above filters) and then exclude any screens within the entire set of data passing the above filters that is extremal relative to this null distribution. As expected, the distribution of $LL4(x_{\min}) - LL4(x_{\max})$ values of those screens that pass the above filters *excluding* the $\delta_{\max}$ filter is shifted to the right relative to those screens that pass all of the above filters *including* the $\delta_{\max}$ filter (Supplementary Fig. 31). Here, we use a Gaussian distribution to model the null distribution and fit it using the right half of the single peak passing all filters in the OHSU dataset or the right half of the rightmost peak passing all filters in the FIMM dataset. We determined the peak/mean of the Gaussian μ by maximizing the density (of values less than zero). The standard deviation σ of the (full) Gaussian

distribution was then determined using the mean U of points exceeding μ as done previously [5]

$$\sigma = (U - \mu)\sqrt{\frac{\pi}{2}} \,. \tag{S4}$$

As in step 7 above, we define a cutoff in $LL4(x_{\min}) - LL4(x_{\max})$ as the least such value at which the cumulative distribution reaches $1/n$, where n is the number of dose-response curves passing the above seven filters. The $LL4(x_{\min}) - LL4(x_{\max})$ cutoff $[LL4(x_{\min}) - LL4(x_{\max})]^* = 58.0$ in the OHSU dataset (where $n = 21,481$) and $[LL4(x_{\min}) - LL4(x_{\max})]^* = 49.5$ in the FIMM dataset (where $n = 12,230$; Supplementary Fig. 31). The cumulative distribution function is inverted using the `qnorm` function in R.

9. Exclude any sample (not patient) for which RNA-seq expression data are not available.

10. If there are multiple screens per patient (after applying filters one through nine above), keep only that one having the best fit [i.e., the lowest RMSE ($LL3$)]. If there are replicates, we first define the screen's RMSE ($LL3$) as the maximum RMSE ($LL3$) across replicates.

11. If there are multiple samples per patient (after applying filters one through 10 above), keep only that one having the best fit [i.e., the lowest RMSE ($LL3$)]. If there are replicates, we first define the sample's RMSE ($LL3$) as the maximum RMSE ($LL3$) across replicates.

Drug response curve filtering applied in each analysis

Filter number	Filter	Filter column name	Routine analysis	Outlier analysis
1	Exclude non-AML	filter.non.aml	Yes	Yes
2	Exclude curves with >1 truncated sensitivities	filter.num.100s	No	Yes
3	Exclude curves with too few or too many concentration points	filter.num.pts	Yes	Yes
4	Exclude dissimilar samples / screens	filter.dissimilar.replicates	Yes	Yes
5	Exclude curves outside common concentration range	filter.restrict.range	Yes	Yes
6a	Exclude based on $\delta_{\max}$	filter.max.delta	No	Yes
6b	Exclude based on RMSE ($LL4$)	filter.rmse.ll4	No	Yes
7	Exclude based on $\|LL3 - LL4\|_{L1}$	filter.l1.norm.norm	No	Yes
8	Exclude based on $LL4(x_{\min}) - LL4(x_{\max})$	filter.left.minus.right.asymptote	No	Yes
9	Exclude based on lack of expression data	filter.expr	Yes	Yes
10	Exclude based on multiple screens per patient	filter.redundant.screen	Yes	Yes
11	Exclude based on multiple samples per patient	filter.redundant.sample	Yes	Yes

Visualizing drug correlations

Individual correlations were plotted with `geom_smooth` in R package `ggplot2` using `method='lm'` and other arguments default, which displays a linear regression fit and a 95% confidence interval. Pairwise correlations are shown as a heatmap created with the `Heatmap`, `HeatmapAnnotation`, and `rowAnnotation` functions in the R `ComplexHeatmap` package. Correlations passed to `Heatmap` where calculated using the R function `cor` and arguments `use='pairwise.complete.obs'` and `method='pearson'`.

Area under the drug response curve

We calculated the Area Under the drug response Curve (AUC) by integrating the sensitivity curve in $\log_{10}$ space from the minimum $x_{\min}$ to the maximum $x_{\max}$ concentration and by normalizing by the width in $\log_{10}$ space of this concentration range. Hence, a larger AUC indicates a sample's higher sensitivity to the drug. For the $LL3$ fits the integral

$$AUC = \frac{1}{\log_{10}(x_{\max}) - \log_{10}(x_{\min})} \int_{x_{\min}}^{x_{\max}} LL3(x) d \log_{10}(x)$$

was computed using `computeAUC` from the `PharmacoGx` R library. Although the `logLogisticFunction` used for fitting is parameterized by viabilities, the integral computed by `computeAUC` is parameterized by sensitivities = 1 - viabilities, as stated in the documentation and confirmed in the source code. A similar integral involving an $LL4$ fit was calculated analytically using supplemental Eq. 1 and Eq. 3 provided by Yadav and colleagues [6]. The resulting (concentration range-normalized) AUC is analogous to DSS_1 with the minimum activity $t = 0$ [6]. The normalized integral involving an $L4$ fit was calculated numerically via adaptive quadrature using the `integrate` function.

We compared the robustness of AUC and EC_{50} values across different models (namely, Eqs. S1, S2, and S3). We found that AUC values were more consistent across models in both the OHSU and FIMM datasets and that this held true when evaluated across all drug response curves (without restriction to a subset of drugs or to AML samples) and when limited (for visualization purposes) to those having: (1) EC_{50} values within the tested drug concentration range, (2) EC_{50} values whose absolute value is within the 95th percentile, (3) and non-negative AUCs (Supplementary Figs. 1 and 2). In the full datasets, EC_{50} values were uncorrelated between $LL4$ and $L4$ fits (OHSU: $r = 1.88 \times 10^{-5}$; FIMM $r = 2.41 \times 10^{-5}$) and between $LL4$ and $LL3$ fits (OHSU: pearson correlation $r = 0.02$; FIMM $r = -1.67 \times 10^{-3}$), while AUCs were highly correlated between $LL4$ and $L4$ fits (OHSU: $r = 0.99$; FIMM; $r = 0.99$) and between $LL4$ and $LL3$ fits (OHSU: $r = 0.85$; FIMM: $r = 0.91$). The following 95th percentiles were used in the scatterplots (OHSU $LL4$: 41849.70, $L4$: 14694.05, $LL3$: 10^6; FIMM $LL4$: 40951.53, $L4$: 17649.42, $LL3$: 65714.22).

Correlation of GRD with clinical characteristics

We tested the association of GRD with patient response to standard induction therapy and with patient survival. For these purposes, we defined GRD on a per-patient basis rather than on the per-sample basis used when modeling (against sample-specific expression data) and in heatmap visualizations. We first removed drug fits of non-AML patients, with fewer than the expected number of concentration points, or with dissimilar replicates (all as described above), but retained fits of drugs assayed in patients without accompanying expression data (either at the sample or patient level) or assayed as biological or technical replicates within a patient. After this filtering step, we confirmed that each individual drug was assayed over a consistent concentration range within (but not necessarily across) datasets. We then defined the patient-level GRD by first averaging unnormalized response AUCs for each patient / drug combination across any biological or technical replicates and then by averaging these across all 87 drugs in common between the OHSU and FIMM datasets for each patient.

Analysis was restricted to the OHSU dataset using published clinical annotations [7], as similar annotations were not available for the FIMM dataset. Patient 2429 in the dataset was removed from the analysis because this individual was associated with multiple samples having inconsistent overall survival times (151 days and 255 days). Patients were further limited to those with AML (`dxAtSpecimenAcquisition=="ACUTE MYELOID LEUKAEMIA (AML) AND RELATED PRECURSOR NEOPLASMS"`) that were treated with standard induction chemotherapy (`typeInductionTx == "Standard Chemotherapy"`). We further restricted patients to those refractory to induction therapy (`responseToInductionTx == "Refractory"`) or achieving a complete response (CR; `responseToInductionTx == "Complete Response"`) or a complete response with incomplete hematologic recovery (CRi; `responseToInductionTx == "Complete Response i"`) to standard induction therapy. Finally, we excluded patients unannotated for overall survival, vital status, or cause of death.

The association between GRD and patient response to induction therapy (CR/CRi versus refractory) was tested via a two-sided Wilcoxon rank sum test using `wilcox.test`.

We tested association between GRD and patient survival by: (1) right censoring long follow-up times, (2) fitting a Cox proportional hazards model, and (3) confirming that the proportional hazards assumption was not violated, as explained in more detail below.

We right censored long follow-up times so their distribution would reflect those expected in a clinical trial in which patients are enrolled uniformly throughout the trial period. This motivation follows that used in performing pooled analyses over multiple clinical trials [8, 9]. Specifically, since patients enter the trial at different dates, at any particular date they will also have different follow-up times. We assume that patient accrual is uniform and that the follow-up times of censored patients are also uniform. Hence, we plotted right-censored (i.e., alive) patients according to increasing survival times and visually observed that the distribution became non-uniform at 610 days (Supplementary Fig. 9).

Patients were right-censored at this time point (i.e., their overall survival time was set to 610 days and their vital status and cause of death were set to alive).

We further restricted the above patient cohort criteria to those with vital status annotated as alive or dead. We dichotomized these patients into a "High GRD" group having a GRD in the top quartile and a "Low GRD" group having a GRD in the bottom quartile. We then performed a Cox proportional hazards analysis with survival as the response variable (i.e., a `Surv` object with time parameter the overall survival and event parameter the GRD group) and GRD group as the independent variable. We fit the model using `coxph` from the `survival` R library. Finally, we confirmed that there was no evidence that the proportional hazards assumption was violated. Specifically, we applied `cox.zph`, which found no evidence that the Schoenfeld residuals were time dependent ($p = 0.48$).

Expression data post-processing

We assessed potential outliers through principal component analysis (PCA) of the [$\log_2$(CPM)] expression data. We did so by applying `prcomp` (with default arguments) to an expression matrix whose columns were genes and rows were samples. OHSU samples 14-00800 and 20-00062 were outliers in a bi-variate plot displaying the first two principal components of the OHSU expression data. These samples had relatively poor alignment quality metric values, including number of aligned reads ($<$ 6th percentile), and were excluded from analysis.

As expected, in a combined analysis of the OHSU and FIMM datasets, the first principal component explained a large proportion of the variance (29%) and separated the two datasets. We corrected for this batch effect by applying `ComBat` from the `sva` R package and confirmed it was removed by again plotting the first two principal components [10, 11].

Gene filtering

Expressed and highly variable genes were used as input for downstream modeling. Genes were first subsetted to those that were expressed independently across two datasets and subsequently into those that were highly variable in the two datasets. Significantly, the two datasets used, OHSU (used for training in downstream analyses) and TCGA AML, were independent of any datasets used for model validation. Genes identified as expressed in OHSU were highly concordant with those identified as expressed in TCGA. Gene expression variability in RNA-seq data has previously been shown to vary smoothly as a function of mean gene expression [12, 13]. According to this mean-variance trend line, lowly-expressed genes are expected to have higher variability (in log space) than highly-expressed genes. This reflects the large contribution in lowly-expressed genes of technical variability (owing to counting statistics) to total variability, which additionally includes biological variation. Modeling should focus on biological variation. Further, those genes with extreme and consistent (across datasets) biological variation are most likely to be robust biomarkers driving

phenotypes (i.e., *ex vivo* drug response in this study). Hence, the degree of biological variation was defined as the residual between the observed and predicted variation (positive residual indicates observed variation is greater than predicted variation) and expressed genes were prioritized according to this variation residual. Variation residuals were highly concordant across OHSU and TCGA datasets. The intersection of expressed genes (in both the OHSU and TCGA datasets) with largest (positive) residuals in both the OHSU and TCGA datasets were those used for downstream modeling. Technical details follow.

RNA-seq counts for TCGA AML data were obtained using the R library `TCGAbiolinks`. Specifically, a query was defined with the command `GDCquery(project = "TCGA-LAML", data.category = "Transcriptome Profiling", experimental.strategy = "RNA-Seq", data.type = "Gene Expression Quantification", workflow.type = "HTSeq - Counts", legacy = FALSE)`, the query was downloaded via `GDCdownload`, and prepared with `GDCprepare`. Results were extracted via `assay` from R library `SummarizedExperiment` using argument `"HTSeq - Counts."` Throughout this manuscript, TCGA data are always subsetted to those AML cases selected here.

RNA-seq read counts for the OHSU and TCGA datasets were converted to counts per million (in $\log_2$ space) via `cpm` with default parameters in R library `edgeR`.

Outlier samples detected above (via PCA) were excluded from the OHSU dataset for subsequent gene filtering (and downstream modeling). Within each dataset, samples had consistent gene expression profiles (Supplementary Fig. 14), further indicating that outliers were properly removed. As expected, profiles in all samples in both datasets had a large density peak at low expression values and a discernible (though more modest) "rightmost" density peak at high expression. Samples in the TCGA dataset also exhibited intermediate peaks. The low expression peak represents lowly- or un-expressed genes. The rightmost peak, with to-be-determined mean/location μ, was interpreted as the profile of robustly-expressed genes, as prior analysis of RNA-seq cancer profiles has shown that it reflects biologically-relevant genes associated with an active promoter as distinguished from genes whose expression falls below μ, represent technical or biological noise, and are associated with repressed promoters [5]. Following that work, a Gaussian distribution was fit to the rightmost peak at μ after first excluding its left half (less than μ) whose boundary is ill defined (particularly in the TCGA dataset) and, hence, would bias estimation of the Gaussian standard deviation parameter σ. This was done by first determining the location of rightmost density peak μ as the point at which the first derivative in the density changes sign (i.e., in principle, the slope should be positive before the peak, zero at the peak, and negative past the peak): the density was approximated between minimum (0) and maximum (10) expression values (using `density` in R), a spline was fit to that approximated density (using `splinefun`), and the location of the rightmost peak μ was taken as the least x value having a first derivative (evaluated by passing `deriv=1` to the fit spline) whose sign differs from the first derivative of its neighbor). The related prior approach that defined zFPKM [5] (and used in filtering drug-response data above) determined the peak of the rightmost peak μ by simply maximizing the density. This would

not have been directly applicable to the OHSU and TCGA datasets, since the rightmost peak μ is not a global maximum. Hence, the described derivative approach (guided by the minimum and maximum boundaries), was more robust in these datasets. The standard deviation σ of the (full) Gaussian distribution was then determined using the mean U of points exceeding μ as done previously [5] using Eq. S4. zFPKM was previously defined as

$$zFPKM = \frac{\log_2(FPKM) - \mu}{\sigma}$$

and a threshold value of zFPKM = -3 was found to discriminate between genes associated with active or repressed chromatin states in ENCODE cell line data. Rather than dichotomize genes as expressed or unexpressed within a sample, we defined a continuous, quantitative summary measure of expression across samples within a dataset for each gene, showed that this measure is consistent across datasets, and finally thresholded this measure to describe genes expressed in AML generally.

To define the gene expression summary measure, we began by defining the probability that each gene was expressed in a given sample. Assuming that the rightmost peak represents expressed genes allowed us to define the probability that a gene with [$\log_2$(CPM)] expression x is expressed as the p-value

$$\int_{-\infty}^{x} N(x'; \mu, \sigma) dx'$$

where $N(x'; \mu, \sigma)$ is the normal distribution with mean μ and standard deviation σ evaluated at x' (Supplementary Figs. 15A-C). We summarized these per-sample p-values across samples by defining their empirical cumulative distribution function (ECDF) across samples within a dataset (Supplementary Figs. 15D-F). We then used the area under the ECDF (ECDF AUC) to characterize the cross-sample expression of a gene. Genes with consistently high expression across samples (i.e., high probabilities of being expressed) have low ECDF AUC (i.e., near zero; Supplementary Figs. 15A,D), those with consistently low expression across samples (i.e., low probabilites of being expressed) have high ECDF AUC (i.e., near one; Supplementary Figs. 15C,F), and those with intermediate expression have correspondingly intermediate ECDF AUC (Supplementary Figs. 15B,E). ECDF AUC was approximated by computing the ECDF by passing a gene's per-sample p-values to the R function `ecdf`, evaluating the returned function at each point in the sequence from 0 to 1 in steps of 0.001, and finally dividing the sum of function evaluated at the points by the length of the sequence.

ECDF AUC values were highly correlated between the OHSU and TCGA datasets (Pearson correlation $r = 0.95$; $p < 2.2\times10^{-16}$; Supplementary Fig. 16). Further, ECDF AUC values were strongly peaked near one in both datasets, suggesting a large set of genes are consistently unexpressed across AML datasets. To establish a dataset-specific cutoff between expressed and unexpressed genes, we plotted the ECDF AUC histograms (margins of Supplementary Fig. 16)

at greater resolution (Supplementary Fig. 17). Both datasets showed similar trends consisting of three linear phases: a first phase (starting at zero ECDF AUC) in which frequency increases with ECDF AUC, a second phase in which frequency increases more gradually with ECDF AUC, and a final phase in which frequency increases sharply with ECDF AUC. We used the breakpoint between the second and third phases to differentiate between expressed genes to the left of the breakpoint (having small AUCs that are not obviously distinguishable from one another) and unexpressed genes to the right of the breakpoint (having large AUCs that rapidly diverge from those AUCs to the left). To determine an objective cutoff, we applied piecewise regression: we calculated the histogram of ECDF AUC values using 100 bins (using `hist` in R with `breaks=100` and `probability=TRUE`), excluded the top five bins with largest density, fit a linear regression model with density as the response and the mid-point of the ECDF AUC bins as the predictor (using `lm` in R), and segmented the regression with initial breakpoint estimates at 0.5 and 0.8 [using `segmented` from the R library `segmented` with `psi=c(0.5, 0.8)`] (Supplementary Fig. 17). This resulted in similar cutoffs for the two datasets (OHSU: 0.86; TCGA: 0.87). The overlap between genes identified as expressed (below the cutoff; OHSU: 10,614 genes; TCGA: 10,754 genes) versus unexpressed (above the cutoff) in both datasets was highly significant (Fisher's exact test $p < 2.2 \times 10^{-16}$). Both results reflect the consistency in relative level of expression of genes across the two datasets. We defined as AML-expressed genes as those 9,805 genes that were expressed in both datasets.

We next subsetted the AML-expressed genes to those that were highly variable. Often highly-variable genes are selected as those with largest standard deviation (of expression across samples) or largest coefficient of variation (CV, i.e., standard deviation normalized by the mean). However, neither of these approaches account for the observation in RNA-seq data that a gene's variation in expression (e.g., the square root of the standard deviation across samples in log space) is smoothly and inversely related to its mean expression (in log space and across samples) [12, 13]. To account for this, we fit a smooth (LOESS or locally estimated scatterplot smoothing) regression curve to model the square root of the standard deviation across samples of gene expression [$\log_2$(CPM)] as a function of the mean across samples of gene expression [$\log_2$(CPM)]. The dip in the trendline at low (mean) expression values (Supplementary Figs. 18A,D) disappeared when genes were limited to those expressed in AML (Supplementary Figs. 18B,E), which revealed the expected and previously-observed trend with lowly-expressed genes having high variation (in log space; i.e., fractional variation in real space) and highly-expressed genes having low variation. We assumed that the majority of genes do not exhibit exceptional variation that would drive phenotypic differences and that this "expected" variation is predicted by the mean-variance trend line. Hence, we focused on those genes with extreme variation by first calculating their residual variation relative to the trend line.

Residual variation was highly correlated between the OHSU and TCGA datasets (Pearson correlation $r = 0.86$; $p < 2.2 \times 10^{-16}$; Supplementary Fig. 19A). We defined a cutoff (see below) in residual variation independently in each

dataset, such that AML-expressed genes above the cutoff were deemed highly variable. This resulted in similar cutoffs for the two datasets (OHSU: 0.09; TCGA: 0.11). This approach does not bias towards selection of lowly-expressed genes (with high variation) as does making a cutoff based on standard deviation (or CV) alone (Supplementary Figs. 18C,F). The overlap between AML-expressed genes identified as highly variable (above the cutoff; OHSU: 2,811 genes; TCGA: 2,551 genes) versus those having stable expression (below the cutoff) in both datasets was highly significant (Fisher's exact test $p < 2.2 \times 10^{-16}$). As observed above with expression, these results reflect the consistency in relative level of variation of genes across the two datasets. We considered those AML-expressed genes identified as highly variable in both datasets as highly variable in AML and used them for downstream modeling ($n = 2,132$; Supplementary Table 3).

The LOESS trend line was fit using `loess(y ~ x, span=0.3, degree=1, family="symmetric", iterations=4, surface="direct")` in R, where x is the mean calculated across samples of gene expression [$\log_2$(CPM)] and y is the square root of standard deviation across samples of gene expression [$\log_2$(CPM)]. Residuals were calculated by passing the result fit object to the `residuals` function.

Cutoffs in residual variation were established based on the 1-standard deviation contour centered at the point of maximal density in the two-dimensional space defined by the residual variation values in both datasets. As these dimensions are highly correlated, the contour is ellipsoidal. To define that ellipse, we rotated the original space defined by the OHSU and TCGA residual variations (Supplementary Fig. 19A) into the space defined by the first two principal components of the residual variations (Supplementary Fig. 19B). This simplifies the definition of the contour as the dimensions are orthogonal in this space. To prevent the heavy tails (particularly in the PC1 dimension) from biasing the contour, we restricted the domain to those points less than the point of maximum density in both dimensions (Supplementary Fig. 19C), fit a (univariate) Gaussian distribution to those points and defined its 1-standard deviation contour about the point of maximal density (Supplementary Fig. 19D), and, finally, rotated the 1-standard deviation contour back into the original space and defined the dataset-specific cutoffs as the maximal points along the contour and in the corresponding dimension (OHSU: 0.09; TCGA: 0.11; Supplementary Fig. 19E).

The above was done as follows: A matrix M was defined with two columns, holding the residual variations in each of the two datasets. We defined a standardized matrix M_s (i.e., with columns of zero mean and unit standard deviation) via `scale` (with arguments `scale=TRUE` and `center=TRUE`) and defined the scale matrix S as a diagonal matrix whose elements are the inverse of the scale factors returned by `scale` (in the `scaled.center` attribute). We performed a principal component analysis of the scaled matrix M_s using `prcomp` (with arguments `scale=FALSE` and `center=FALSE`), which returns the matrix $M_{r,s}$ of (scaled) data elements rotated from the original space into the principal component space in the returned value `x` and the rotation matrix R that

performs this operation in the returned value `rotation`, i.e., $M_{r,s} = M_s R$. The inverse rotation (i.e., that rotates data from the principal component to the original space) is performed by the inverse matrix R^{-1}, which, for a rotation matrix, is simply the transpose R^T: $M_s = M_{r,s}R^{-1} = M_{r,s}R^T$. Rotation into this principal component space decouples the x and y axes of the original space such that the covariance matrix of the Gaussian distribution describing the data in the original space is diagonalized in the principal component space–i.e., univariate Gaussian distributions can be fit to the rotated data. We calculated the point of maximal density $(\mu_{\mathrm{PC1}}, \mu_{\mathrm{PC2}})$ of the rotated data $M_{r,s}$ using kernel density estimation (i.e., by passing its columns to the R function `kde2d` in library `MASS` and specifying that 200 grid points should be used in the estimation with `n=200`) and used μ_{PC1} and μ_{PC2} as the centers/means of univariate Gaussian distributions whose standard deviations σ_{PC1} and σ_{PC2} were calculated via Eq. S4. These two univariate distributions are equivalent to the bivariate Gaussian distribution with mean vector $\mu_{r,s} \equiv (\mu_{\mathrm{PC1}}, \mu_{\mathrm{PC2}})^T$ and diagonal covariance matrix $\Sigma_{r,s}$ with elements σ^2_{PC1} and σ^2_{PC2}. Given the transformation $M = M_s S^{-1} = M_{r,s}R^{-1}S^{-1}$, we transform the covariance matrix back into the original (unscaled) space via

$$\begin{aligned}\Sigma &= \mathbb{E}[M^T M] = \mathbb{E}[S^{-T}R^{-T}M^T_{r,s}M_{r,s}R^{-1}S^{-1}] \\ &= S^{-T}R^{-T}\mathbb{E}[M^T_{r,s}M_{r,s}]R^{-1}S^{-1} = S^{-T}R^{-T}\Sigma_{r,s}R^{-1}S^{-1} \ .\end{aligned}$$

As in the transformed space, we use the point of maximal density $\mu \equiv (\mu_x, \mu_y)$ of the data M in the original space determined via `kde2d` as the mean/center of a bivariate Gaussian distribution. We calculate 1-standard deviation contours (i.e., encompassing 68% of the data) using `ellipse` in library `mixtools` with arguments `npoints=200` and `alpha=1-(pnorm(1)-pnorm(-1))`. To calculate the contour in the original space we pass `ellipse` μ and Σ, whereas to calculate the contour in the transformed space we pass it $\mu_{r,s}$ and $\Sigma_{r,s}$.

Drug response and GRD modeling

As described above, modeling was restricted to a single sample (assayed for both drug response and gene expression) per patient. Each drug was independently modeled. For modeling, drug response AUC, GRD, and gene expression $\log_2$(CPM) values were standardized–i.e., each drug (or the GRD) was transformed so as to have zero mean response and unit standard deviation and, similarly, each gene was transformed to have zero mean expression and unit standard deviation.

Observed GRD was modeled using gene expression only. Observed GRD for patient sample s is the mean over drug d of the unnormalized $AUC_{d,s}$, i.e., $(1/n_s)\sum_d AUC_{d,s}$, where n_s is the number of drugs assayed for sample s. Drugs included in the sum are those 87 drugs in common between OHSU and FIMM.

Drug response was modeled using four sets of features: (1) gene expression only; (2) observed GRD only; (3) gene expression and observed GRD; (4) gene expression and predicted GRD. So as to avoid circularity and over-fitting, the

observed GRD when used as a feature in modeling drug d is calculated by excluding d from the mean, i.e., as $[1/(n_s - 1)] \sum_{d' \neq d} AUC_{d',s}$. Similarly, the predicted GRD when used as a feature in modeling drug d is the prediction in the FIMM dataset of the model trained on the observed GRD calculated without drug d in the OHSU dataset.

Ridge regression models were fit using `cv.glmnet` from the `glmnet` R package, using parameters `family = "gaussian"`, `type.measure = "mse"`, `nfolds = 5`, `standardize = FALSE` (since standardization is performed prior to calling `cv.glmnet`, as described above), `alpha = 0` (to specify ridge regression), and `intercept = FALSE` (to exclude an intercept from the model). Observed or predicted GRD was not penalized when included as a feature along with gene expression. This was accomplished by defining a vector with an entry for each feature, setting that entry to zero if it corresponds to GRD and to one otherwise, and passing this vector as the `penalty.factor` argument of `cv.glmnet`. Ridge predictions were extracted from a fitted model by passing it to `predict` with argument `s = lambda.1se`. Features and their associated weights were extracted from a fitted model by passing it to `coefficients` along with argument `s = lambda.1se`.

Bayesian multi-source regression

BMSR models drug response based on the expression of N_G genes according to

$$\mathbf{y}^{(d)} \sim N(X^{(d)}\boldsymbol{\beta}^{(d)}, \sigma^{(d)}\mathbf{I}) \; , \tag{S5}$$

where $\mathbf{y}^{(d)} \in \mathbb{R}^{N_d \times 1}$ is the response vector for a particular drug across the N_d patient samples in dataset $d \in \{\text{FIMM}, \text{OHSU}\}$, $X^{(d)} \in \mathbb{R}^{N_d \times N_G}$ is the corresponding expression matrix over N_G genes, $\boldsymbol{\beta}^{(d)} \in \mathbb{R}^{N_G \times 1}$ is the gene regression coefficient vector, and $\mathbf{I}$ is the $N_G \times N_G$ identity matrix. Since the expression of each gene was z-transformed to zero mean and unit variance, we do not include an intercept term in the model. The standard deviation $\sigma^{(d)}$ is assumed to have the non-informative noise prior

$$\sigma^{(d)} \sim IG(1, 1) \; ,$$

where $IG(\alpha, \beta)$ is the Inverse Gamma distribution[1]

$$IG(x; \alpha, \beta) = \frac{\beta^\alpha}{\Gamma(\alpha)} (1/x)^{\alpha+1} e^{-\beta/x}$$

and $\Gamma(\cdot)$ is the Gamma function.

BMSR performs joint regression across the two datasets by modeling the coefficient vector $\boldsymbol{\beta}^{(d)}$ using the joint hierarchical prior

$$\boldsymbol{\beta}^{(d)} \sim N(\boldsymbol{\beta}, 0.5)$$

[1] https://mc-stan.org/docs/2_21/functions-reference/inverse-gamma-distribution.html

parameterized by the shared mean coefficient vector $\boldsymbol{\beta} \in \mathbb{R}^{N_G \times 1}$. Each of its components β_g corresponding to gene g is regularized using the Finnish horse-shoe prior [14]

$$\begin{aligned}\beta_g &\sim N(0, \lambda_g^2 \tau^2)\\ \lambda_g &\sim C^+(0,1)\\ \tau &\sim C^+(0, \tau_0)\\ \tau_0 &= \frac{p_0}{N_G - p_0} \frac{\sum_d \sigma^{(d)}}{\sqrt{N_d}} \ ,\end{aligned}$$

where $C^+(\mu, \sigma)$ is the half-Cauchy distribution with location μ and scale σ. The scalar λ_g induces localized gene-wise regularization and the scalar τ is the global regularization parameter that induces the number of active genes (p_0) *a priori*. The Cauchy distribution is defined as[2]

$$C(x; \mu, \sigma) = \frac{1}{\pi\sigma} \frac{1}{1 + [(x-\mu)/\sigma]^2} \ .$$

BMSR is implemented using the R interface to the STAN programming language [15]. Model inference was performed using STAN's implementation of Hamiltonian Monte Carlo Sampler [15] with 500 samples of the posterior preceded by a burnin of another 500 iterations. The model was run for a single chain, with STAN's `adapt_delta` set to a high value (0.999) to avoid divergences. We used 5 fold-cross validation to select the optimal value of the user-defined hyper-parameter $p_0 = [5, 20, 100]$, which tunes the *a priori* number of genes for prediction. The optimal value of p_0 was identified as the one with minimal cross-validation root mean squared error of the predicted responses.

The BMSR-predicted (scalar) response $y_p^{(d)}$ for a patient p with expression vector $\mathbf{x}_p^{(d)}$ in dataset d was calculated as the expected value of the posterior predictions, i.e., by averaging over the S posterior sample vectors $\boldsymbol{\beta}_s^{(d)}$

$$y_p^{(d)} = \frac{1}{S} \sum_{s=1}^{S} \mathbf{x}_p^{(d)} \cdot \boldsymbol{\beta}_s^{(d)} \ ,$$

where $\cdot$ is the dot product. The coefficient vectors $\widehat{\boldsymbol{\beta}^{(d)}}$ used for identifying predictive biomarkers in dataset d and displayed in **Figs. 3** and **5** are similarly calculated as averages over posterior samples

$$\widehat{\boldsymbol{\beta}^{(d)}} = \frac{1}{S} \sum_{s=1}^{S} \boldsymbol{\beta}_s^{(d)} \ .$$

The Finnish horse-shoe prior encourages the dataset-specific coefficients $\widehat{\boldsymbol{\beta}^{(d)}}$ to either have large magnitude in both datasets (i.e., representing genes whose

[2] https://mc-stan.org/docs/2_21/functions-reference/cauchy-distribution.html

expression makes a large contribution to the response) or small magnitude in both datasets (i.e., genes with little or no contribution) and to have the same direction (i.e., sign) in both datasets.

BMSR is available at https://github.com/suleimank/bmsr.

Bayesian multi-source multi-task regression

Bayesian multi-source multi-task regression (BMSMTR) simultaneously analyzes both multiple datasets (multi-source) and multiple drugs (multi-task) in the set of drugs $\mathcal{I}$. It does so by generalizing Eq. S5 according to

$$\mathbf{y}^{(d,i)} \sim N\left(X^{(d)}\boldsymbol{\beta}^{(d)}w^{(i)}, \sigma^{(d)}\mathbf{I}\right) ,$$

with the response vector $\mathbf{y}^{(d,i)} \in \mathbb{R}^{N_d \times 1}$ for dataset d and drug $i \in \mathcal{I}$ distributed about a mean that is a product of a factor $X^{(d)}\boldsymbol{\beta}^{(d)}$ common to drugs in $\mathcal{I}$ and a factor $w^{(i)} \sim N(0.5, 0.5)$ specific to drug i.

The additional computational time required of BMSMTR relative to BMSR was mitigated by reducing the number of input gene features. Amongst the 9,805 AML-expressed genes, we selected the top 500 genes with highest residual variation in the each of the OHSU and TCGA datasets. There was a large overlap between the two datasets, with 609 genes selected and used as input features to BMSMTR.

BMSMTR is available at https://github.com/suleimank/bmsr.

Feature overlap across models

Overlap between ridge model features trained independently on the OHSU and FIMM datasets was calculated as a function of the number of top features considered n_{top}. Specifically, independently in each dataset we sorted the n_{genes} genes according to the magnitude of their ridge model coefficient. We then calculated n_{overlap} as the number of genes amongst the highest magnitude n_{top} genes in both datasets additionally having coefficients with the same sign in both models. This was plotted against the number of genes expected to overlap $n_{\text{expected overlap}}$ using a hypergeometric model, i.e., $(n_{\text{top}}^2)/(2n_{\text{genes}})$, where the 2 in the denominator accounts for the fact that a gene can have a positive or non-positive coefficient.

Gene set enrichment analysis

Gene set enrichment analysis (GSEA) was performed using the `fgsea` function in the `fgsea` R package [16]. Enrichment was performed on a vector `stats`, where the ith component of `stats` is the average coefficient of feature i across ridge models independently trained on FIMM and OHSU using gene expression features. Enrichment was performed independently with respect to the gene ontology (GO) [17, 18], Hallmark, Biocarta, KEGG, Reactome, and monocyte genes defined by CIBERSORT [19]. For GO, Entrez gene identifiers were associated

with GO terms using `org.Hs.egGO2ALLEGS` from the `org.Hs.eg.db` R library and then translated to symbols using `org.Hs.egSYMBOL`. The mapping between GO terms and gene symbols was uploaded to Synapse and is accessible with Synapse ID `syn20641475`. Hallmark [20], BioCarta (https://cgap.nci.nih.gov/Pathways/BioCarta_Pathways), and KEGG [21], and Reactome [22] Gene Matrix Transposed (GMT) files were downloaded from MSigDG [23] at the listed URLs, processed using `gmtPathways`, and uploaded to Synapse where they are accessible with the listed identifiers:

Gene sets used in analysis

Gene Set	URL	Synapse ID
Hallmark	http://software.broadinstitute.org/gsea/msigdb/download_file.jsp?filePath=/resources/msigdb/6.0/h.all.v6.0.symbols.gmt	syn10507487
Biocarta	http://software.broadinstitute.org/gsea/msigdb/download_file.jsp?filePath=/resources/msigdb/6.0/c2.cp.biocarta.v6.0.symbols.gmt	syn10507483
KEGG	http://software.broadinstitute.org/gsea/msigdb/download_file.jsp?filePath=/resources/msigdb/6.0/c2.cp.kegg.v6.0.symbols.gmt	syn10507485
Reactome	http://software.broadinstitute.org/gsea/msigdb/download_file.jsp?filePath=/resources/msigdb/6.0/c2.cp.reactome.v6.0.symbols.gmt	syn10507486

For purposes of GSEA, monocyte genes were those defined as differentially expressed in monocytes relative to the other 21 leukocyte populations examined by the CIBERSORT deconvolution method [19]. fgsea was run with the parameters `minSize = 2`, `maxSize = 1000`, and `nperm = 10000`.

Defining monocytic signature

We defined a monocytic signature of venetoclax response by (1) identifying venetoclax biomarkers using BMSR, (2) limiting these to genes associated with monocytes, and (3) compressing the expression of these genes into a single score using gene set variation analysis (GSVA) [24].

We identified venetoclax biomarkers by combining the BMSR coefficients across the FIMM and OHSU datasets for each gene using Stouffer's method. Specifically, we translated the BMSR coefficients into z-scores independently for each dataset–i.e., such that they have zero mean and unit standard deviation. We then calculated the combined z-score z_i for gene i using its z-scores $z_{i,d}$ across datasets: $z_i = \frac{1}{\sqrt{2}} \sum_{d \in \{\text{FIMM,OHSU}\}} z_{i,d}$. Finally, we calculated the p-value of z_i using the standard normal distribution function (i.e., using `pnorm` with default parameters) and selected as venetoclax biomarkers those with a nominal (uncorrected) p-value < 0.01.

We defined monocyte-associated genes as those that were differentially expressed ($p < 0.05$) between monocytes and more than half of the following 18 populations derived from umbilical cord or peripheral blood [GSE24759 [25]]: CD4+ central memory, CD4+ effector memory, CD8+ central memory, CD8+ effector memory, CD8+ effector memory RA, early B cell, mature B cells capable of class switching, mature class-switched B cells, mature B cells, mature CD45+CD16+CD3- NK cells, mature CD56-CD16+CD3- NK cells, mature CD56-CD16-CD3- NK cells, NKT cells, naive CD4+ T cells, naive CD8+ T cells, naive B cells, plasmacytoid dendritic cells, and pro B cells. Differential expression was determined using the functions `lmFit`, `eBayes`, and `topTable` within the `limma` R package, all invoked with default parameters [26, 27].

These two steps identified the following monocyte-associated venetoclax biomarkers: $BCL3$, $CD14$, $LILRB1$, $LRP1$, $MAFB$, $PSAP$, $SLC15A3$, and $SLC7A7$. We manually confirmed that each of these genes were highly expressed in monocytes relative to other stem and hematopoietic progenitor populations [GSE42519 [28], accessed using the online BloodSpot tool [29]]. We defined the monocytic signature by compressing the expression of these genes into a single score using the `gsva` function from the `GSVA` R package with default parameters.

Supplementary References

[1] Tang, J. *et al.* Drug Target Commons: A Community Effort to Build a Consensus Knowledge Base for Drug-Target Interactions. *Cell Chem Biol* **25**, 224–229 (2018).

[2] Ritz, C., Baty, F., Streibig, J. C. & Gerhard, D. Dose-Response Analysis Using R. *PLoS ONE* **10**, e0146021 (2015).

[3] Smirnov, P. *et al.* PharmacoGx: an R package for analysis of large pharmacogenomic datasets. *Bioinformatics* **32**, 1244–1246 (2016).

[4] Safikhani, Z. *et al.* Revisiting inconsistency in large pharmacogenomic studies. *F1000Res* **5**, 2333 (2016).

[5] Hart, T., Komori, H. K., LaMere, S., Podshivalova, K. & Salomon, D. R. Finding the active genes in deep RNA-seq gene expression studies. *BMC Genomics* **14**, 778 (2013).

[6] Yadav, B. *et al.* Quantitative scoring of differential drug sensitivity for individually optimized anticancer therapies. *Sci Rep* **4**, 5193 (2014).

[7] Tyner, J. W. *et al.* Functional genomic landscape of acute myeloid leukaemia. *Nature* **562**, 526–531 (2018).

[8] Sargent, D. J. *et al.* Disease-free survival versus overall survival as a primary end point for adjuvant colon cancer studies: individual patient data from 20,898 patients on 18 randomized trials. *J. Clin. Oncol.* **23**, 8664–8670 (2005).

[9] Sargent, D. *et al.* Two or three year disease-free survival (DFS) as a primary end-point in stage III adjuvant colon cancer trials with fluoropyrimidines with or without oxaliplatin or irinotecan: data from 12,676 patients from MOSAIC, X-ACT, PETACC-3, C-06, C-07 and C89803. *Eur. J. Cancer* **47**, 990–996 (2011).

[10] Leek, J. T. *et al.* *sva: Surrogate Variable Analysis* (2016). R package version 3.20.0.

[11] Johnson, W. E., Li, C. & Rabinovic, A. Adjusting batch effects in microarray expression data using empirical Bayes methods. *Biostatistics* **8**, 118–127 (2007).

[12] Law, C. W., Chen, Y., Shi, W. & Smyth, G. K. voom: Precision weights unlock linear model analysis tools for RNA-seq read counts. *Genome Biol.* **15**, R29 (2014).

[13] Love, M. I., Huber, W. & Anders, S. Moderated estimation of fold change and dispersion for RNA-seq data with DESeq2. *Genome Biol.* **15**, 550 (2014).

[14] Piironen, J. & Vehtari, A. Sparsity information and regularization in the horseshoe and other shrinkage priors. *Electron. J. of Stat.* 5018–5051 (2017).

[15] Carpenter, B. *et al.* Stan: A probabilistic programming language. *J. Stat. Softw.* **76** (2017).

[16] Sergushichev, A. A. An algorithm for fast preranked gene set enrichment analysis using cumulative statistic calculation. *BioRxiv* (2016). URL `https://www.biorxiv.org/content/10.1101/060012v1`.

[17] Ashburner, M. *et al.* Gene ontology: tool for the unification of biology. The Gene Ontology Consortium. *Nat. Genet.* **25**, 25–29 (2000).

[18] The Gene Ontology Consortium. The Gene Ontology Resource: 20 years and still GOing strong. *Nucleic Acids Res.* **47**, D330–D338 (2019).

[19] Newman, A. M. *et al.* Robust enumeration of cell subsets from tissue expression profiles. *Nat. Methods* **12**, 453–457 (2015).

[20] Liberzon, A. *et al.* The Molecular Signatures Database (MSigDB) hallmark gene set collection. *Cell Syst* **1**, 417–425 (2015).

[21] Kanehisa, M. & Goto, S. KEGG: kyoto encyclopedia of genes and genomes. *Nucleic Acids Res.* **28**, 27–30 (2000).

[22] Fabregat, A. *et al.* The Reactome Pathway Knowledgebase. *Nucleic Acids Res.* **46**, D649–D655 (2018).

[23] Liberzon, A. *et al.* Molecular signatures database (MSigDB) 3.0. *Bioinformatics* **27**, 1739–1740 (2011).

[24] Hanzelmann, S., Castelo, R. & Guinney, J. GSVA: gene set variation analysis for microarray and RNA-seq data. *BMC Bioinformatics* **14**, 7 (2013).

[25] Novershtern, N. *et al.* Densely interconnected transcriptional circuits control cell states in human hematopoiesis. *Cell* **144**, 296–309 (2011).

[26] Ritchie, M. E. *et al.* limma powers differential expression analyses for RNA-sequencing and microarray studies. *Nucleic Acids Res.* **43**, e47 (2015).

[27] Phipson, B., Lee, S., Majewski, I. J., Alexander, W. S. & Smyth, G. K. Robust Hyperparameter Estimation Protects Against Hypervariable Genes And Improves Power To Detect Differential Expression, journal= .

[28] Rapin, N. *et al.* Comparing cancer vs normal gene expression profiles identifies new disease entities and common transcriptional programs in AML patients. *Blood* **123**, 894–904 (2014).

[29] Bagger, F. O., Kinalis, S. & Rapin, N. BloodSpot: a database of healthy and malignant haematopoiesis updated with purified and single cell mRNA sequencing profiles. *Nucleic Acids Res.* **47**, D881–D885 (2019).

Supplementary Figures

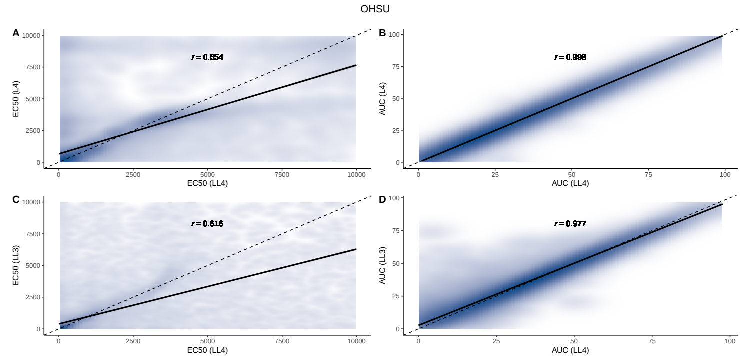

Supplementary Figure 1: AUC is more robust than EC_{50} in OHSU dataset. Density plot comparing (A) EC_{50} values from 4-parameter log-logistic ($LL4$) and 4-parameter logistic ($L4$) fits, (B) AUCs from $LL4$ and $L4$ fits, (C) EC_{50} values from $LL4$ and 3-parameter log-logistic ($LL3$) fits, and (D) AUCs from $LL4$ and $LL3$ fits in OHSU dataset. Solid black line: regression line; dashed black line: identity line. r: Pearson correlation. Plotted values of drug response curves: with $LL4$- and $L4$-derived EC_{50} values between minimum and maximum tested drug concentration and having absolute value less than the 95th percentile and with non-negative $LL4$- and $L4$-derived AUCs (A and B) or with $LL4$- and $LL3$-derived EC_{50} values between minimum and maximum tested drug concentration and having absolute value less than the 95th percentile and with non-negative $LL4$-derived AUCs (C and D).

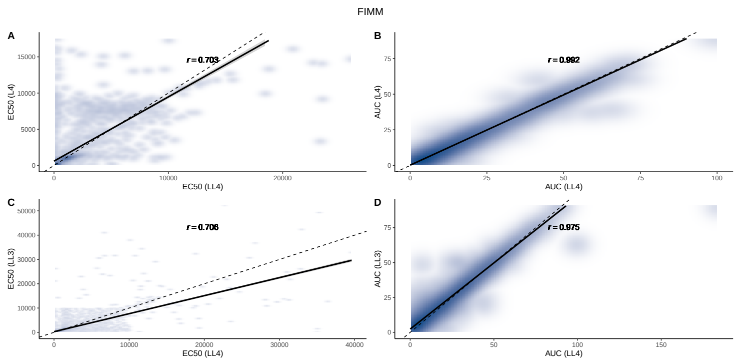

Supplementary Figure 2: AUC is more robust than EC_{50} in FIMM dataset. Density plot comparing (A) EC_{50} values from 4-parameter log-logistic ($LL4$) and 4-parameter logistic ($L4$) fits, (B) AUCs from $LL4$ and $L4$ fits, (C) EC_{50} values from $LL4$ and 3-parameter log-logistic ($LL3$) fits, and (D) AUCs from $LL4$ and $LL3$ fits in FIMM dataset. Solid black line: regression line; dashed black line: identity line. r: Pearson correlation. Plotted values of drug response curves: with $LL4$- and $L4$-derived EC_{50} values between minimum and maximum tested drug concentration and having absolute value less than the 95th percentile and with non-negative $LL4$- and $L4$-derived AUCs (A and B) or with $LL4$- and $LL3$-derived EC_{50} values between minimum and maximum tested drug concentration and having absolute value less than the 95th percentile and with non-negative $LL4$-derived AUCs (C and D).

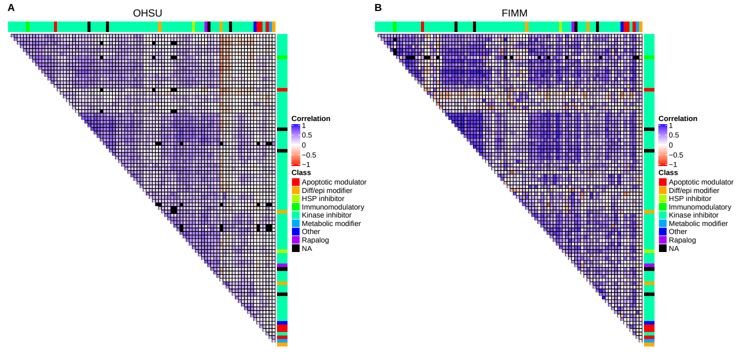

Supplementary Figure 3: Drugs common to both datasets are enriched for kinase inhibitors that are highly correlated. Pairwise drug Pearson correlations across (A) OHSU and (B) FIMM datasets. Drugs ($n = 87$) ordered in both datasets according to hierarchical clustering of drugs in OHSU dataset (complete linkage clustering based on distance defined as 1 - correlation). Class: drug class; Diff/epi: differentiation/epigenetic; HSP: heat shock protein.

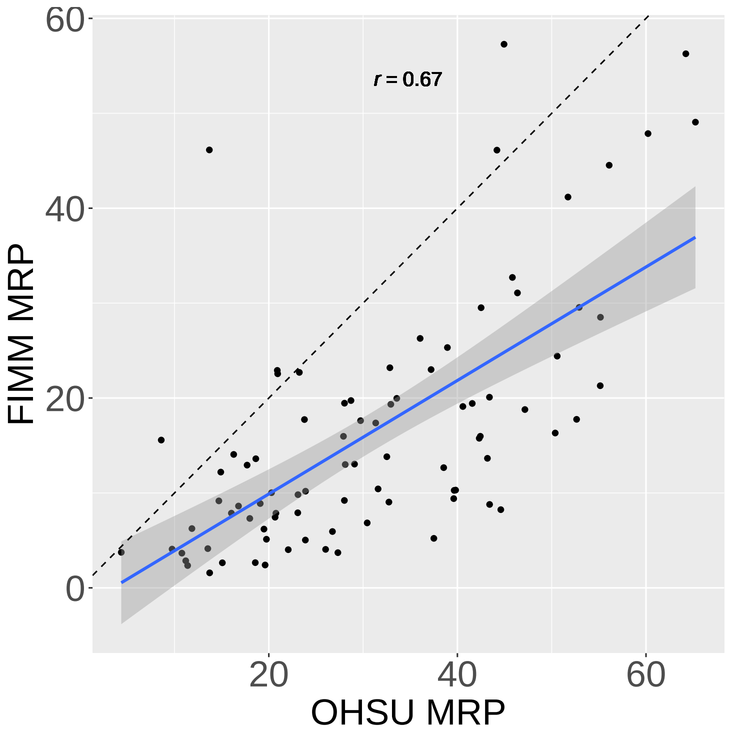

Supplementary Figure 4: Mean response across patients is highly correlated between OHSU and FIMM datasets. Mean response across patients (MRP) for each drug (n = 87) common to the OHSU (x axis) and FIMM (y axis) datasets. MRP is mean of raw AUCs for an individual drug over patients. r: Pearson correlation; dashed line: identity line; blue line: linear regression fit; gray shading: 95% confidence interval.

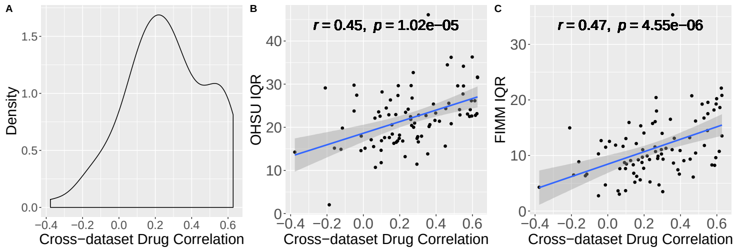

Supplementary Figure 5: Consistency of drug response across datasets is impacted by drug response dynamic range. (A) Density of cross-dataset drug correlation. (B, C) Cross-dataset drug correlation versus interquartile range (IQR) of drug response AUC in (B) OHSU and (C) FIMM datasets. Cross-dataset drug correlation is the correlation of two dataset-specific drug response profiles. The drug response profile for a drug is the vector of correlations of its response with that of response to all other drugs in the dataset. r: Pearson correlation; blue line: linear regression fit; gray shading: 95% confidence interval.

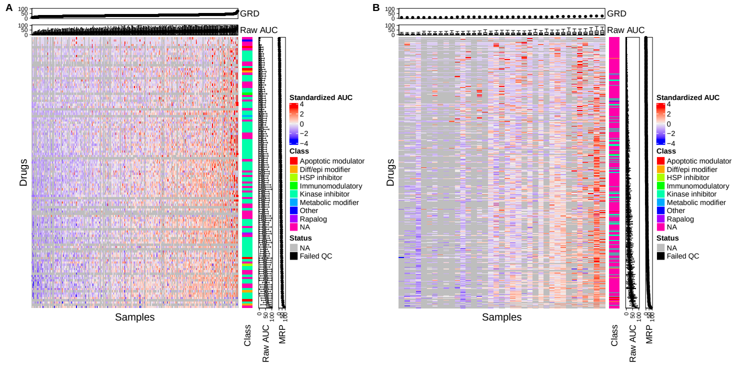

Supplementary Figure 6: A patient's *ex vivo* responses are similar across all drugs. AUCs calculated across all patient-derived *ex vivo* samples (columns) and drugs (rows) in OHSU (A; 338 samples and 122 drugs) and FIMM (B; 37 samples and 470 drugs) datasets. Red values correspond to higher AUC or more sensitive samples, blue are less sensitive, black are filtered, and gray are missing. Standardized AUCs (i.e., with mean zero and standard deviation one across patients) displayed in heatmap. Raw AUCs displayed in top and side panels. General response across drugs (GRD) is mean of raw AUCs for an individual patient over drugs; Mean response across patients (MRP) is mean of raw AUCs for an individual drug over patients. Samples ordered by GRD in each dataset. Drugs ordered by MRP in each dataset. One sample displayed per patient, with sample assayed across highest number of drugs displayed in cases with multiple samples per patient. Class: drug class; Diff/epi: differentiation/epigenetic; HSP: heat shock protein.

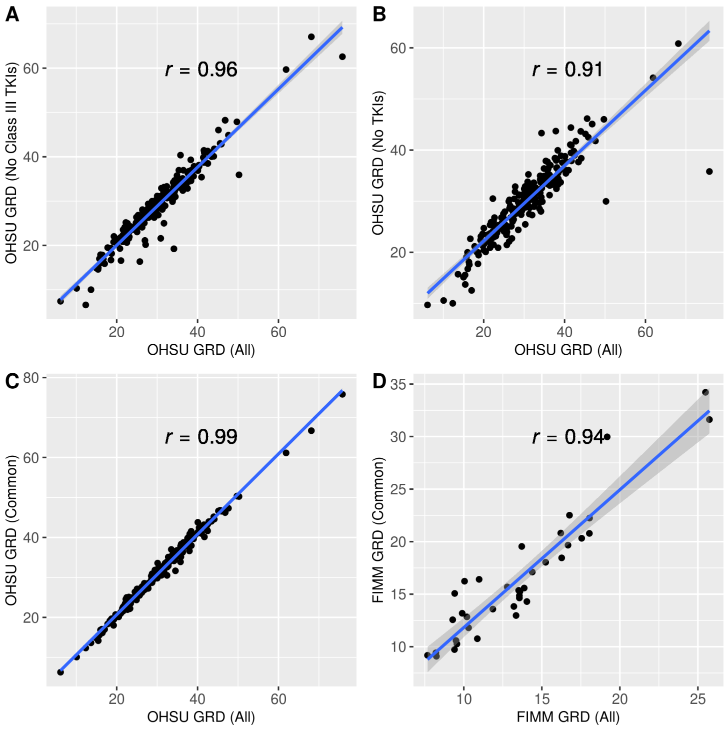

Supplementary Figure 7: General response across drugs is stable across drug set used to compute it. GRD computed across all drugs in the OHSU (A, B, C; n=122) or FIMM (D; n=477) datasets versus that computed from drugs excluding class III TKIs (A; n=97), from drugs excluding all TKIs (B; n=74), or from drugs common to OHSU and FIMM (C, D; n=87). TKI: tyrosine kinase inhibitor. Each point corresponds to a patient. r: Pearson correlation; blue line: linear regression fit; gray shading: 95% confidence interval.

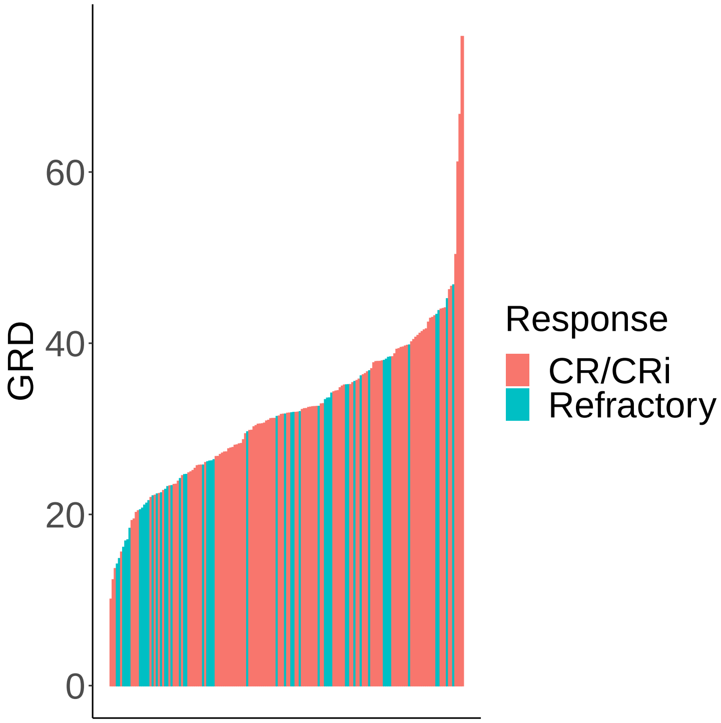

Supplementary Figure 8: Patients achieving complete response to induction chemotherapy are enriched for those with high general response across drugs. General response across drugs (GRD; y axis) of each patient (x axis) in OHSU dataset. Patients ordered by GRD, with indicated response to standard induction chemotherapy. CR/CRi: complete response / complete response with incomplete hematologic recovery.

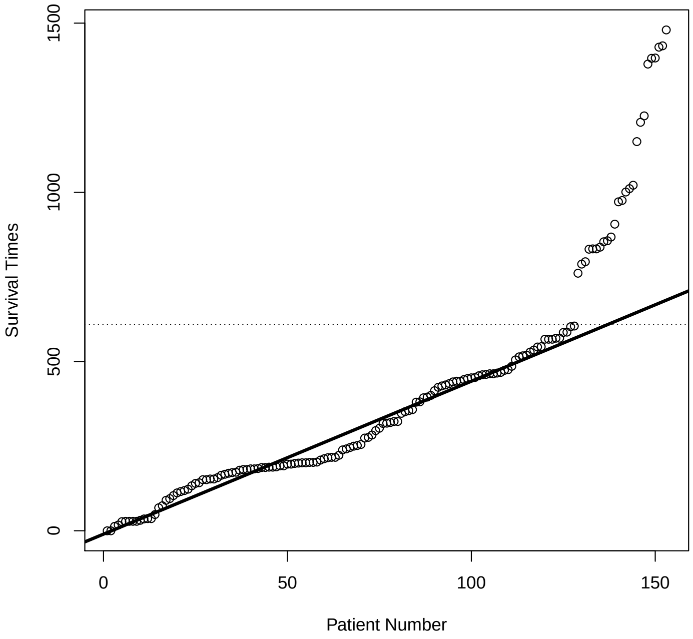

Supplementary Figure 9: Several patients have extreme follow-up times. Overall survival of patients in OHSU dataset who remained alive throughout the study period, ordered by increased survival time. Solid black line demonstrates uniform distribution of follow-up times up to 610 days (indicated by horizontal dotted line).

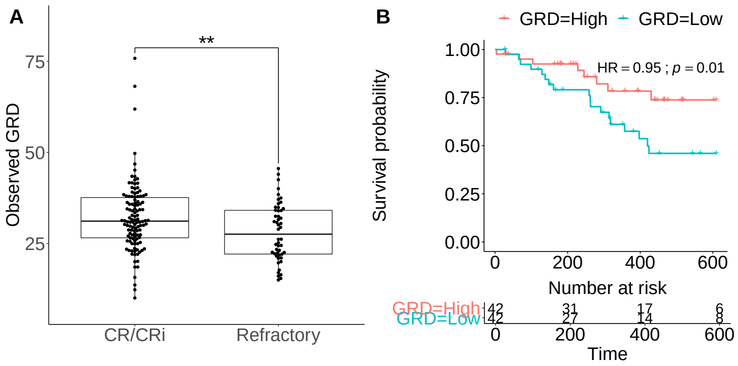

Supplementary Figure 10: *Ex vivo* general response across all drugs is associated with clinical response and improved patient outcome. (A) GRD in patients that achieve complete remission (CR) or complete remission with incomplete hematologic recovery (CRi) to standard induction chemotherapy (n=118) versus those refractory to induction (n=50) in OHSU dataset. **: Wilcoxon rank sum $p < 0.01$. Boxplot indicates median, lower and upper hinges (at first and third quartiles, respectively), lower whisker [at the least value at most 1.5 × IQR (inter-quartile range or distance between first and third quartiles) below the lower hinge] and upper whisker (at the greatest value at most 1.5 × IQR above the upper hinge). (B) Kaplan Meier survival curves of patients in OHSU dataset with GRD above the upper quartile (red; "responders"; n=42) and of those with GRD below the lower quartile (blue; "non-responders"; n=42). Data are right censored at 610 days. GRD is computed across all drugs in OHSU dataset. HR: Cox proportional hazard ratio.

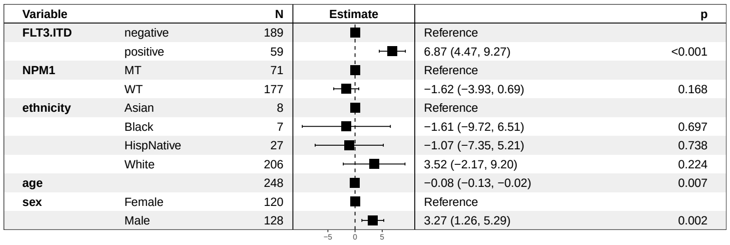

Supplementary Figure 11: *FLT3*-ITD is independently associated with general response across drugs. Multivariate analysis of OHSU dataset. Single patient with AdmixedWhite ancestry excluded from analysis.

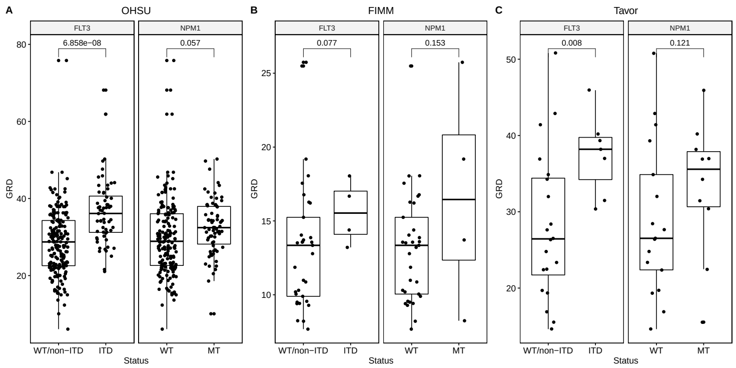

Supplementary Figure 12: *FLT3*-ITD is associated with increased general response across drugs. GRD versus *FLT3* status (WT/non-ITD or ITD) and *NPM1* status (WT or MT) in (A) OHSU, (B) FIMM, and (C) Tavor datasets. Two-sided Wilcoxon signed rank p values in discovery OHSU dataset and one-sided Wilcoxon signed rank p values in FIMM and Tavor datasets. ITD: internal tandem duplication; WT: wild type; MT: mutant. Boxplot indicates median, lower and upper hinges (at first and third quartiles, respectively), lower whisker [at the least value at most $1.5 \times$ IQR (inter-quartile range or distance between first and third quartiles) below the lower hinge] and upper whisker (at the greatest value at most $1.5 \times$ IQR above the upper hinge).

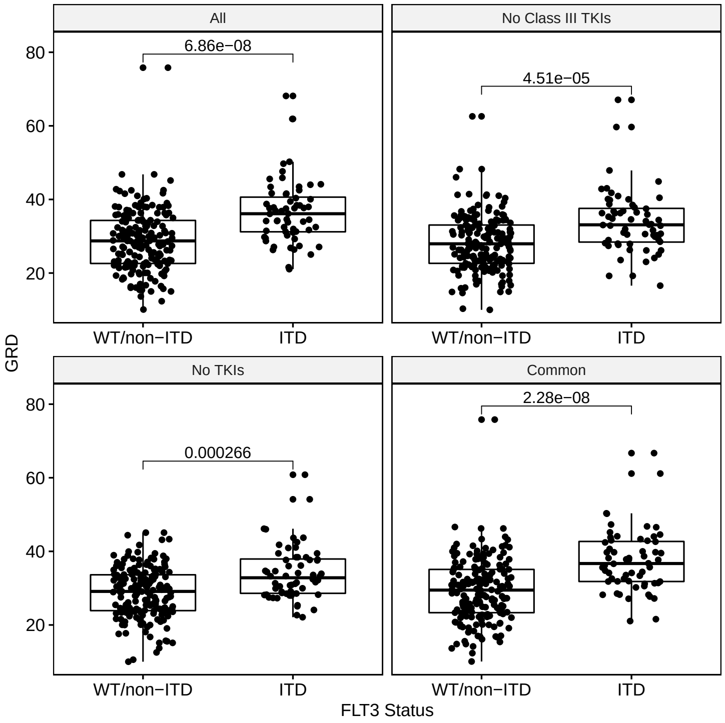

Supplementary Figure 13: *FLT3*-ITD is associated with increased general response across drugs independent of drug set used to compute it. GRD versus *FLT3* status (WT/non-ITD or ITD) and *NPM1* status (WT or MT). GRD computed across all drugs in the OHSU dataset, across drugs excluding class III TKIs, across drugs excluding all TKIs, or across drugs common to OHSU and FIMM. Each point corresponds to a patient. Two-sided Wilcoxon signed rank p values. TKI: tyrosine kinase inhibitor; ITD: internal tandem duplication; WT: wild type; MT: mutant. Boxplot indicates median, lower and upper hinges (at first and third quartiles, respectively), lower whisker [at the least value at most $1.5 \times$ IQR (inter-quartile range or distance between first and third quartiles) below the lower hinge] and upper whisker (at the greatest value at most $1.5 \times$ IQR above the upper hinge).

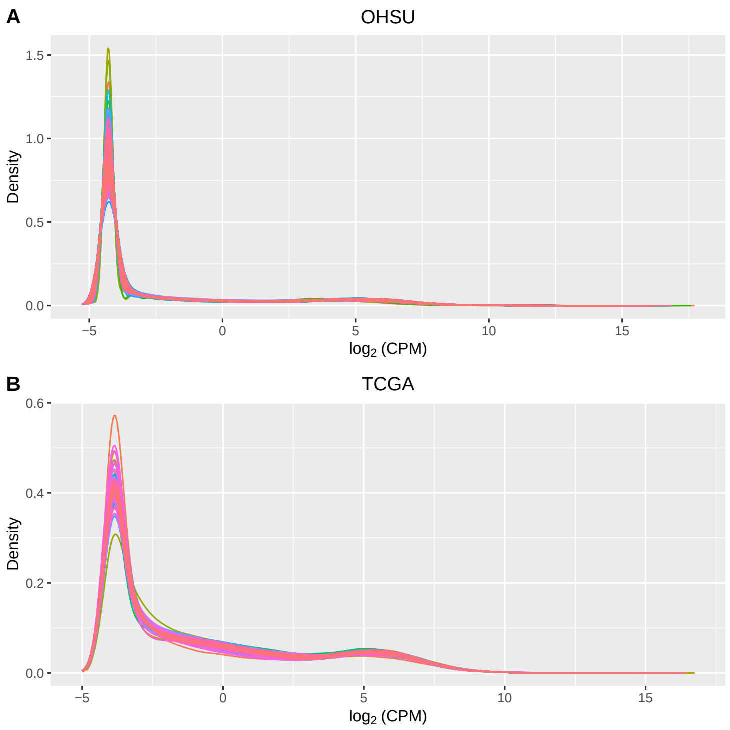

Supplementary Figure 14: Expression distributions are consistent across samples. Density of gene expression [base-2 logarithm of counts per million, $\log_2$(CPM)] in (A) OHSU ($n = 565$) and (B) TCGA ($n = 151$) samples. OHSU samples filtered to exclude outliers detected via PCA.

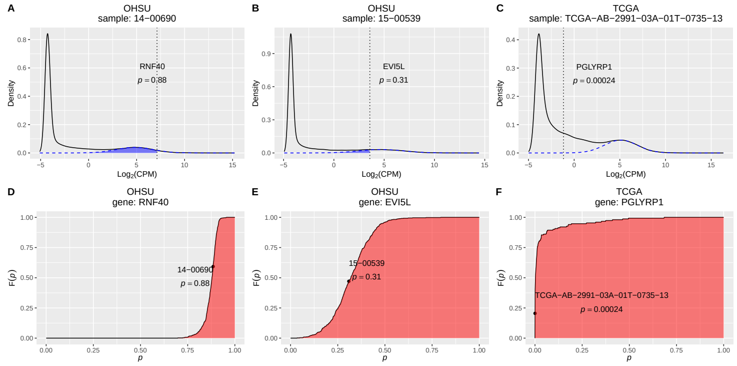

Supplementary Figure 15: Cumulative distribution function of probability that gene is expressed evaluated over samples quantifies gene expression within a dataset. (A-C) Normal distribution (dashed line) fitted to rightmost peak of gene expression [base-2 logarithm of counts per million, $\log_2$(CPM)] density in a sample. Rightmost peak assumed to represent expressed genes; hence probability of a gene being expressed at a given level is p-value of associated expression relative to fitted normal distribution. (D-F) Empirical cumulative distribution function (ECDF) of a gene's p-values across all samples within a dataset. Area under the ECDF curve (ECDF AUC; red shading) quantifies that gene's expression across dataset. Examples shown for highly- (*RNF40*; panels A and D), moderately- (*EVI5L*; panels B and E), and lowly-expressed (*PGLYRP1*; panels C and F) genes. For indicated gene in indicated sample, p-value for that gene's level of expression (x axis; dotted vertical line; panels A-C) in that sample is highlighted in the p-value ECDF across all samples (dot; panels D-F).

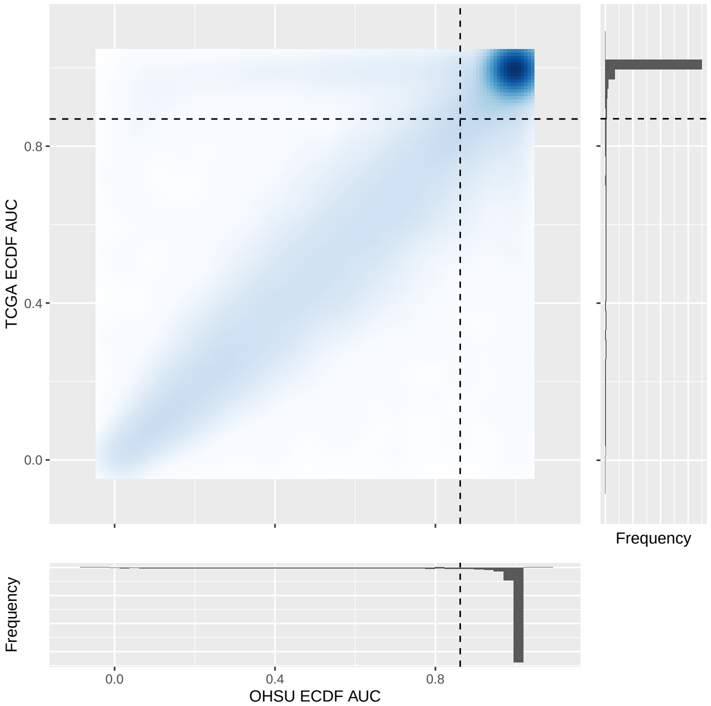

Supplementary Figure 16: Genes are expressed consistently across datasets. Density across genes of ECDF AUCs in both OHSU and TCGA datasets (center plot) and histograms of ECDF AUCs within TCGA (right panel) or OHSU (bottom panel) datasets. Dashed lines: ECDF AUC cutoffs.

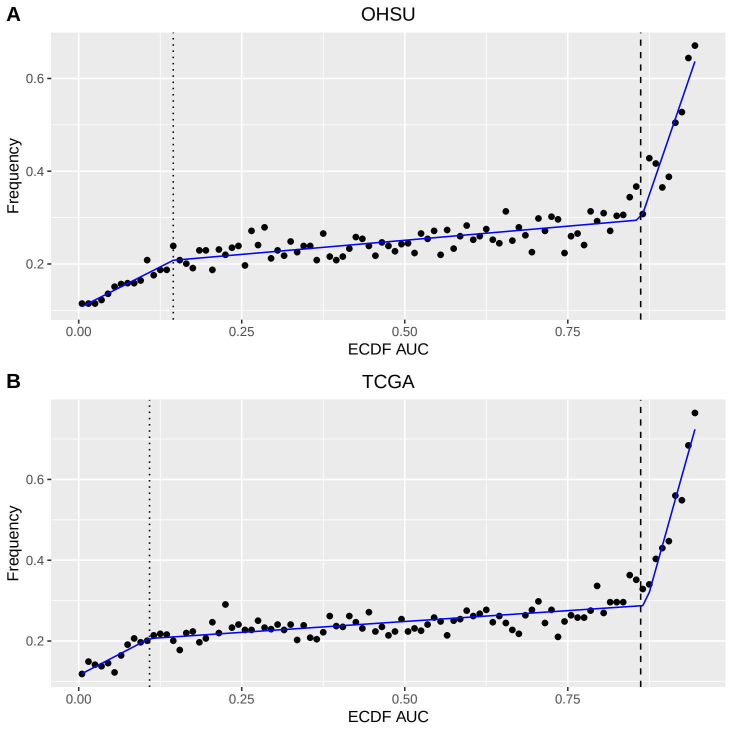

Supplementary Figure 17: Elbow in frequency of ECDF AUCs establishes dataset-specific cutoff between unexpressed and expressed genes. Histogram frequency of ECDF AUCs in (A) OHSU and (B) TCGA datasets. Solid line: piecewise regression fit to frequency of ECDF AUCs with two breakpoints (dotted and dashed vertical lines). Elbow (second breakpoint; dashed vertical line) used as cutoff between unexpressed and expressed genes.

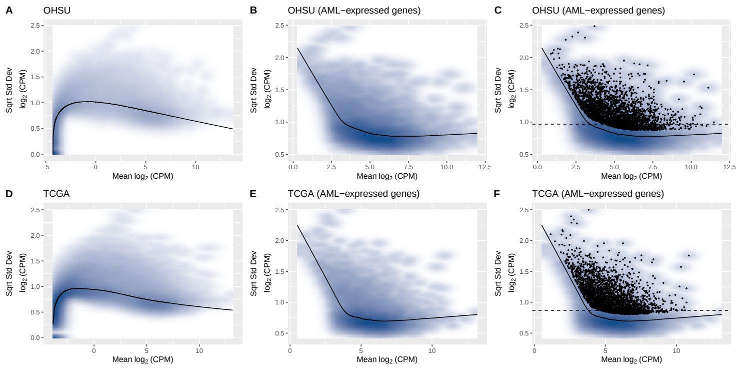

Supplementary Figure 18: Genes used as features are expressed and have high variation above mean-variance trend line. (A,D) Density across genes of mean of and variation in their expression in (A) OHSU and (D) TCGA datasets. Mean calculated across samples in dataset of gene expression [base-2 logarithm of counts per million, $\log_2$(CPM)]. Variation calculated as square root of standard deviation across samples of gene expression [$\log_2$(CPM)]. Solid curve: mean-variance LOESS trend line. (B,E) Density of gene expression mean of and variation in AML-expressed genes in (B) OHSU and (E) TCGA datasets. (C,F) AML-expressed genes identified as highly variable independently in (C) OHSU ($n = 2,811$) and (F) TCGA ($n = 2,551$) datasets. Highly variable is defined relative to mean-variance trend line–i.e., according to residual between observed and trend-predicted variation. The intersection of the two genesets were considered highly-variable, AML-expressed genes ($n = 2,132$) and used for downstream analysis. Genes above dashed line (OHSU: $n = 2,811$; TCGA: $n = 2,551$) would be selected instead based on highest variation (y axis).

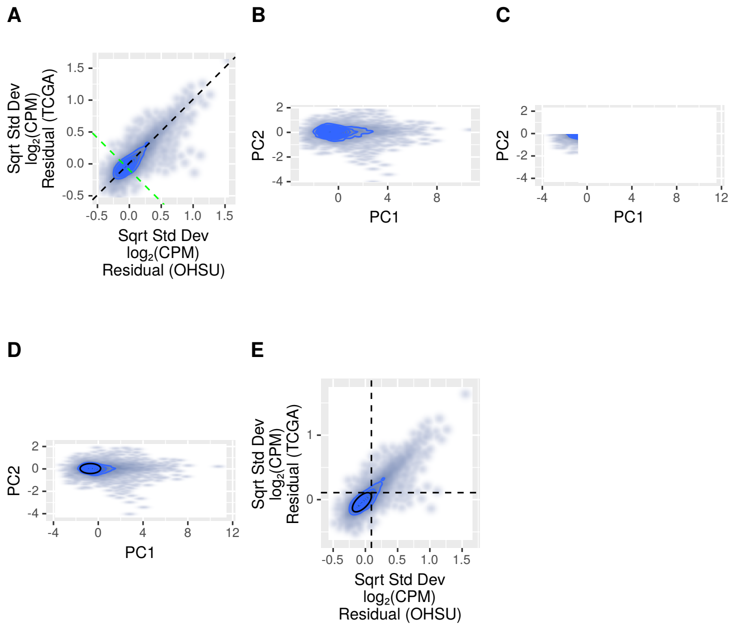

Supplementary Figure 19: Consistency of variation of expressed genes across datasets establishes variation cutoff. (A) Density and contour lines (blue curves) across genes of variation of gene expression in OHSU (x axis) and TCGA (y axis) datasets. Variation calculated as square root of standard deviation across samples of gene expression [$\log_2$(CPM)]. Dashed black line: PC1; Dashed green line: PC2. (B) Density and contour lines of gene variance projected (i.e., rotated) onto PC1 (x axis) and PC2 (y axis). (C) Density and contour lines of gene variation projected onto PC1 and PC2, restricted to values less than empirically-estimated point of maximum density. Univariate normal distributions were fit independently to restricted values independently in each dimension. (D) Overlay of fitted 1-standard deviation contour line from fitted normal distribution (solid black curve) and empirical contour lines (blue curves) on density plot projected onto PC1 and PC2. (E) Overlay of fitted 1-standard deviation contour line from normal distribution (fitted in principal component space and rotated back into linear space; black curve) and empirical contour lines (blue curves) on density plot.

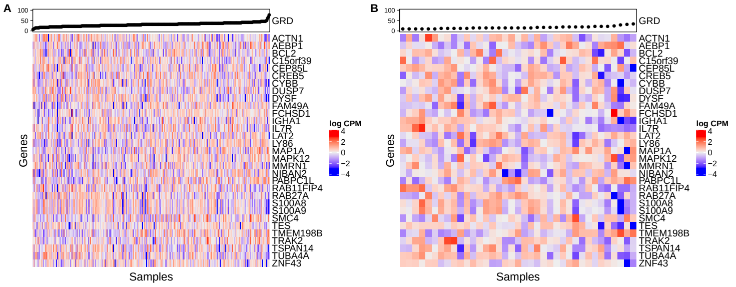

Supplementary Figure 20: Candidate GRD biomarker expression. Expression (standardized base-2 logarithm of counts per million, $\log_2$(CPM)] of BMSR-prioritized GRD biomarkers in (A) OHSU and (B) FIMM datasets. Samples are ordered according to GRD. GRD is computed across drugs common to OHSU and FIMM datasets.

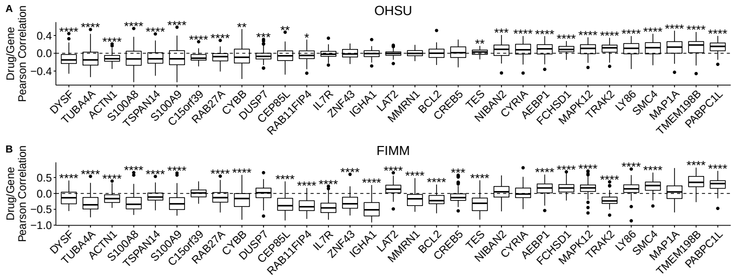

Supplementary Figure 21: BMSR-nominated biomarkers of general response across drugs are consistently correlated with individual drug response across datasets. Distribution of correlation of individual drugs (n=87 common drugs; y axis) from general response across drugs (GRD) signature with indicated gene (x axis) across (A) OHSU and (B) FIMM datasets. Genes are ordered according to their mean (across drugs) drug correlation. ****: Two-sided paired Wilcoxon signed rank Benjamini Hochberg (BH)-adjusted $p < 0.0001$; ***: BH-adjusted $p < 0.001$; **: BH-adjusted $p < 0.01$.

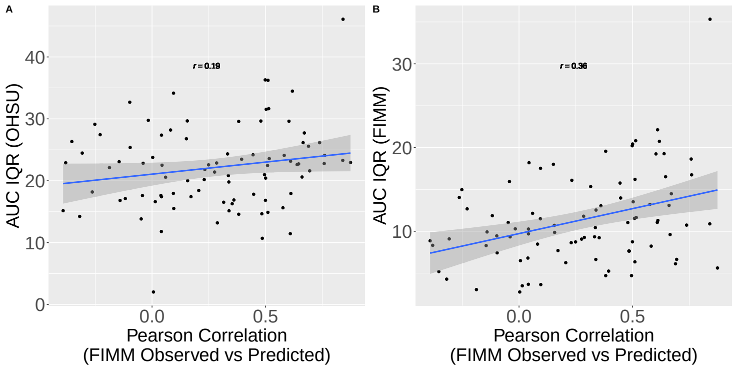

Supplementary Figure 22: Drug prediction is correlated with interquartile range in response. Prediction performance (Pearson correlation of observed and predicted drug response in FIMM dataset; x axis) versus interquartile range (IQR) of drug response AUC in (A) OHSU and (B) FIMM datasets. r: Pearson correlation; blue line: linear regression fit; gray shading: 95% confidence interval.

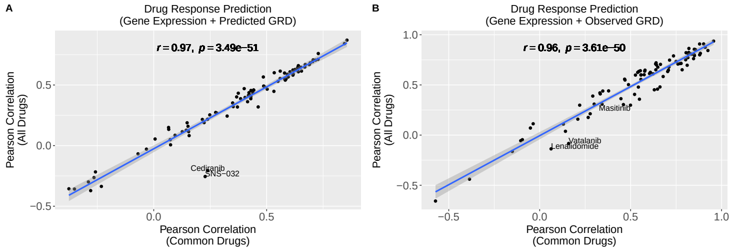

Supplementary Figure 23: Drug response predicted using GRD covariate computed across all drugs is highly correlated with that computed across common drugs. Correlation of drug response observed in FIMM dataset relative to that predicted from OHSU training dataset using GRD as a covariate (A) predicted or (B) observed across all drugs (y axis) or drugs common to FIMM and OHSU (x axis). Each point corresponds to a drug (n=87). r: Pearson correlation; blue line: linear regression fit; gray shading: 95% confidence interval.

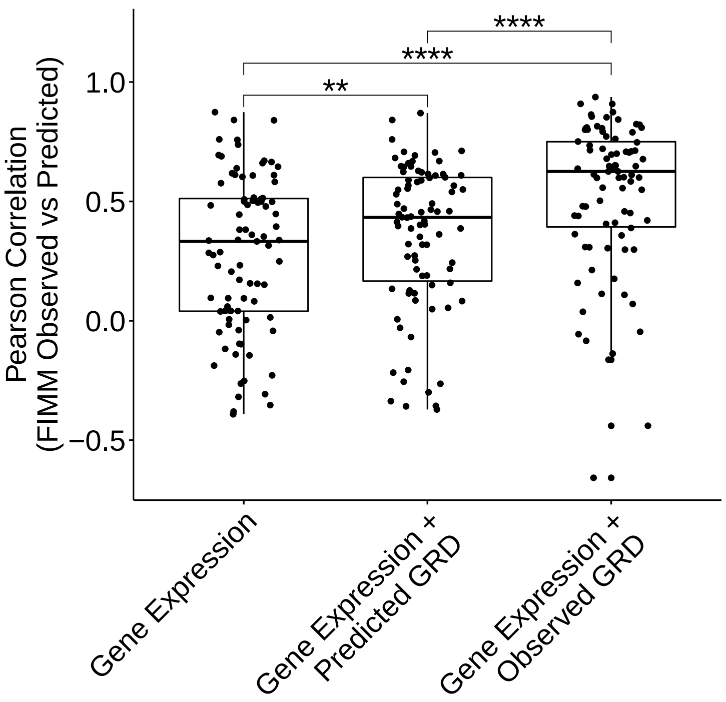

Supplementary Figure 24: Expression-based predictions of drug response indicate concordance of independent ex vivo datasets and may be improved by incorporating general response across drugs (computed across all drugs). Performance (Pearson correlation between observed and model-predicted drug response; y axis) of ridge regression models trained on OHSU data and tested on FIMM data using genes as predictors (Gene Expression), genes and GRD predicted by applying ridge regression to gene expression (Gene Expression + Predicted GRD), or genes and GRD calculated from drug response data (Gene Expression + Observed GRD). Drug d is excluded from observed and predicted GRD in modeling d's response. GRD is computed across all drugs in the OHSU or FIMM dataset, as appropriate. Each point corresponds to a drug (n=87). ****: One-sided paired Wilcoxon signed rank $p < 0.0001$; **: $p < 0.01$. Boxplot indicates median, lower and upper hinges (at first and third quartiles, respectively), lower whisker [at the least value at most $1.5 \times$ IQR (inter-quartile range or distance between first and third quartiles) below the lower hinge] and upper whisker (at the greatest value at most $1.5 \times$ IQR above the upper hinge).

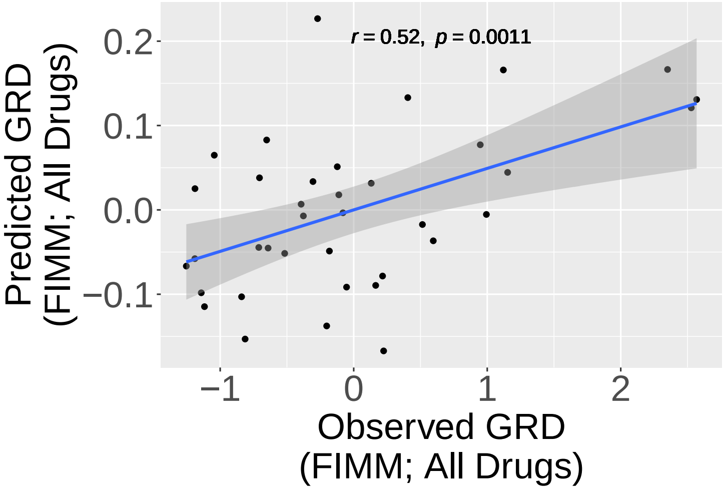

Supplementary Figure 25: Prediction of general response across drugs is impacted by domain of drugs considered. Observed (x axis) versus model-predicted (y axis) GRD, with GRD computed across all drugs. r: Pearson correlation; blue line: linear regression fit; gray shading: 95% confidence interval.

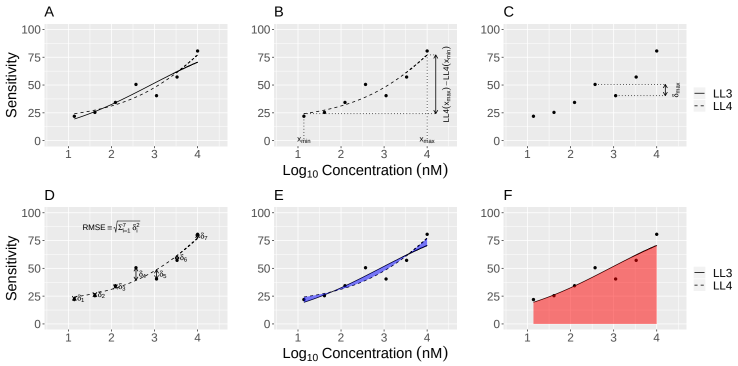

Supplementary Figure 26: Measures quantitatively summarize drug response curves. (A) $LL3$ (solid line) and $LL4$ (dashed line) fits to observed drug sensitivity sensitivity(x) (dots; y axis) as a function of drug concentration x ($\log_{10}$; nM; x axis). (B) Difference in fitted sensitivity, $LL4(x_{\min}) - LL4(x_{\max})$, between $LL4$ fit at minimum ($x_{\min}$) and maximum ($x_{\max}$) concentration points. (C) Maximum change in sensitivity, $\delta_{\max} = \max_i [\text{sensitivity}(x_{i-1}) - \text{sensitivity}(x_i)]$, between neighboring concentration points x_{i-1} and x_i. (D) Root-mean-square error, RMSE $= \sqrt{\sum_i \delta_i^2}$, calculated from residuals $\delta_i = |LL4(x_i) - \text{sensitivity}(x_i)|$ between $LL4$ fit and observed drug sensitivity. (E) Integral of absolute difference in log space between $LL3$ and $LL4$ fits, $\|LL3 - LL4\|_{L1} = \int_{x_{\min}}^{x_{\max}} |LL3(x) - LL4(x)| d\log_{10}(x)$ (blue shading). (F) Area under the $LL3$ curve calculated in log space, $AUC = \int_{x_{\min}}^{x_{\max}} LL3(x) d\log_{10}(x)$ (red shading).

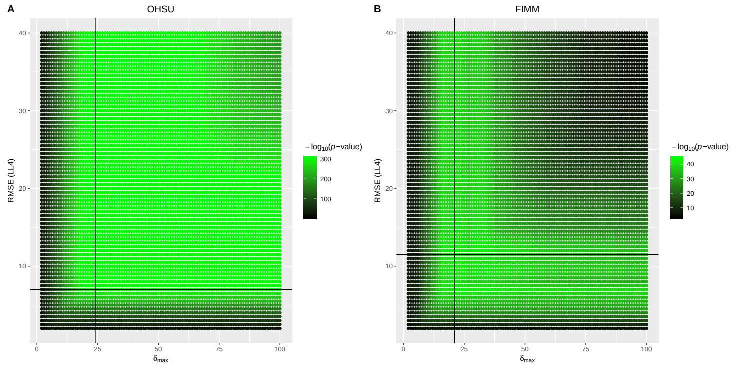

Supplementary Figure 27: Intra-replicate root-mean-square difference establishes optimal cutoffs of quality of fit metrics RMSE ($LL4$) and $\delta_{\max}$. Heatmap of values [$-\log_{10}(p)$; Wilcoxon rank sum test p-value] indicating the significance in differences between the distribution of intra-replicate root-mean-square differences (RMSDs) of those drug response curves passing a quality of fit filter [i.e., with $\delta_{\max}$ less than the value on the x axis and RMSE ($LL4$) less than the value on the y axis] and the distribution of RMSDs of those drug response curves excluded by that filter. Black lines indicate optimal cutoffs (i.e., that minimize the p-value) $\delta^*_{\max}$ of $\delta_{\max}$ (vertical line) and RMSE $(LL4)^*$ of RMSE ($LL4$) (horizontal line) for (A) OHSU [$\delta^*_{\max} = 24$ and RMSE $(LL4)^* = 7$] and (B) FIMM [$\delta^*_{\max} = 21$ and RMSE $(LL4)^* = 11.5$] datasets. RMSDs calculated between responses of two technical replicates for OHSU and of two biological replicates for FIMM.

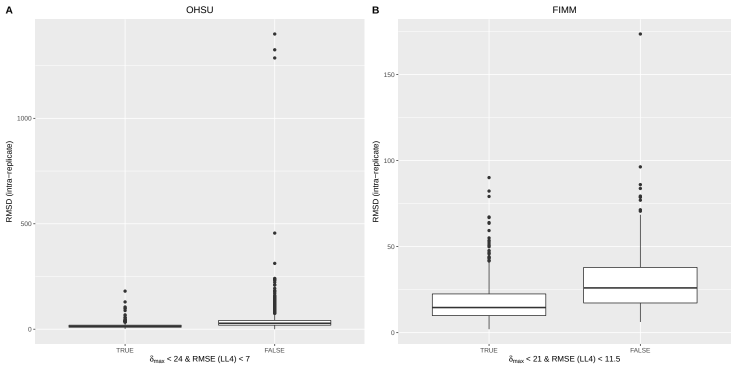

Supplementary Figure 28: Optimal cutoffs of quality of fit metrics RMSE ($LL4$) and $\delta_{\max}$ segregate drug response curves according to intra-replication variance. Intra-replicate root-mean-square differences (RMSDs; y axis) of drug response curves that do or do not pass optimized quality of fit filter (x axis) in (A) OHSU or (B) FIMM datasets. RMSDs calculated between responses of two technical replicates for OHSU dataset and of two biological replicates for FIMM dataset. Boxplot indicates median, lower and upper hinges (at first and third quartiles, respectively), lower whisker [at the least value at most $1.5 \times$ IQR (inter-quartile range or distance between first and third quartiles) below the lower hinge] and upper whisker (at the greatest value at most $1.5 \times$ IQR above the upper hinge).

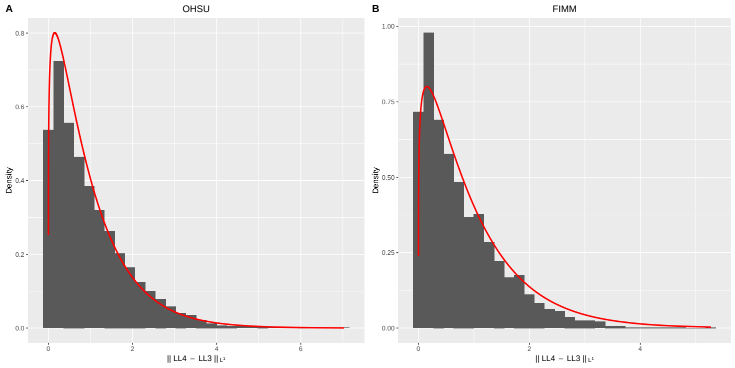

Supplementary Figure 29: Gamma distributions characterize empirical $\|LL3 - LL4\|_{L1}$ distributions. Gamma distribution (red curve) fit to $\|LL3 - LL4\|_{L1}$ density (gray bar) for (A) OHSU and (B) FIMM datasets.

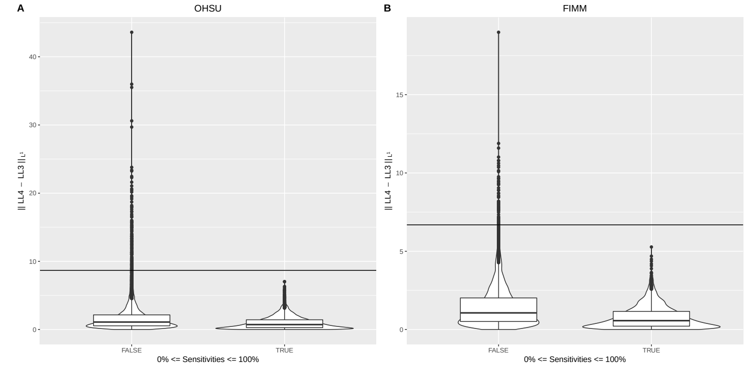

Supplementary Figure 30: Bounded sensitivity range establishes optimal cutoff of quality of fit metric $\|LL3 - LL4\|_{L1}$. $\|LL3 - LL4\|_{L1}$ values (y axis) for those drug response curves having all drug sensitivity values that do or do not all lie between 0 and 100% (x axis) in the (A) OHSU and (B) FIMM datasets. Boxplot indicates median, lower and upper hinges (at first and third quartiles, respectively), lower whisker [at the least value at most $1.5\times$IQR (inter-quartile range or distance between first and third quartiles) below the lower hinge] and upper whisker (at the greatest value at most $1.5 \times$ IQR above the upper hinge). Points indicate outliers. Black horizontal line indicates optimal cutoff $\|LL3-LL4\|^*_{L1}$ of $\|LL3-LL4\|_{L1}$ (OHSU: $\|LL3-LL4\|^*_{L1} = 8.7$; FIMM: $\|LL3 - LL4\|^*_{L1} = 6.7$). Boxplot indicates median, lower and upper hinges (at first and third quartiles, respectively), lower whisker [at the least value at most $1.5 \times$ IQR (inter-quartile range or distance between first and third quartiles) below the lower hinge] and upper whisker (at the greatest value at most $1.5\times$IQR above the upper hinge).

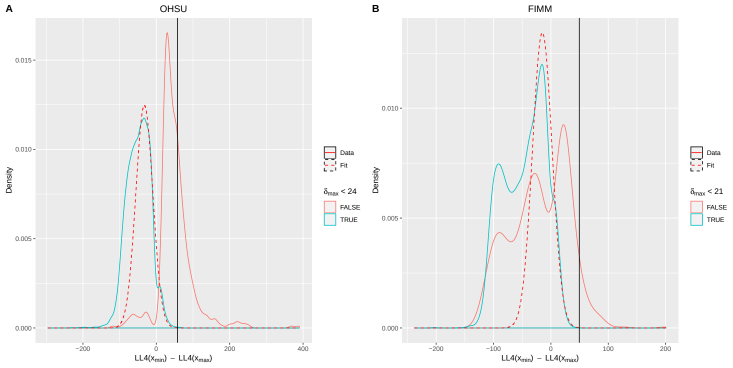

Supplementary Figure 31: Tail of distribution establishes optimal cutoff of quality of fit $LL4(x_{\min}) - LL4(x_{\max})$. Density of $LL4(x_{\min}) - LL4(x_{\max})$ for those drug response curves passing (blue; $\delta_{\max} < \delta^*_{\max}$) or failing (red; $\delta_{\max} \geq \delta^*_{\max}$) the $\delta_{\max}$-filter for the (A) OHSU ($\delta^*_{\max} = 24$) and (B) FIMM ($\delta^*_{\max} = 21$) datasets. Fit of Gaussian distribution (dashed curve) to single (OHSU) or right-most (FIMM) peak passing $\delta_{\max}$-filter. Black vertical line indicates optimal cutoff $[LL4(x_{\min}) - LL4(x_{\max})]^*$ of $LL4(x_{\min}) - LL4(x_{\max})$ (OHSU: $[LL4(x_{\min}) - LL4(x_{\max})]^* = 58.0$; FIMM: $[LL4(x_{\min}) - LL4(x_{\max})]^* = 49.5$).

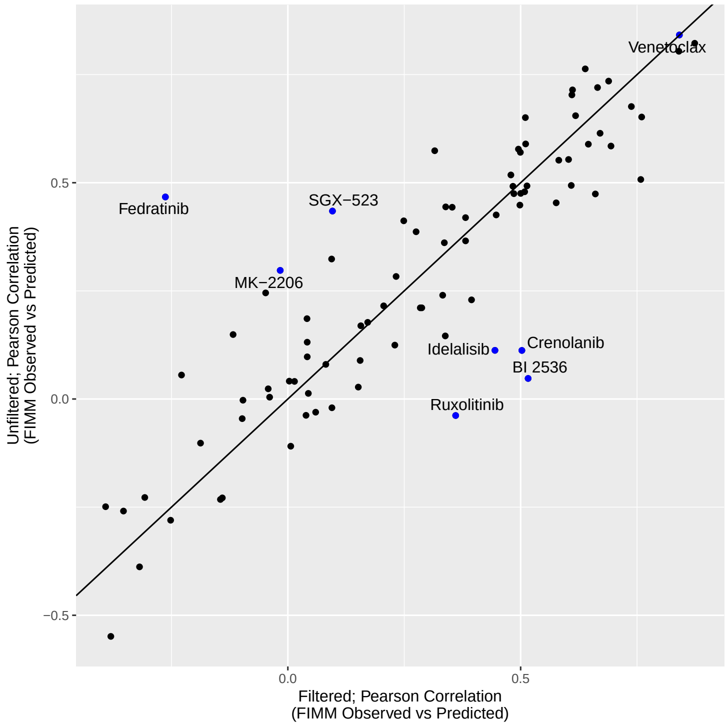

Supplementary Figure 32: Filtering of drug data does not affect prediction performance for most drugs. Prediction performance (Pearson correlation of observed and predicted drug response in FIMM dataset using ridge regression trained on OHSU gene expression data) of filtered (x axis) on unfiltered (y axis) drug response data. Labeled drugs include venetoclax and those having a difference in performance between a filtered and unfiltered analysis greater than 0.3.

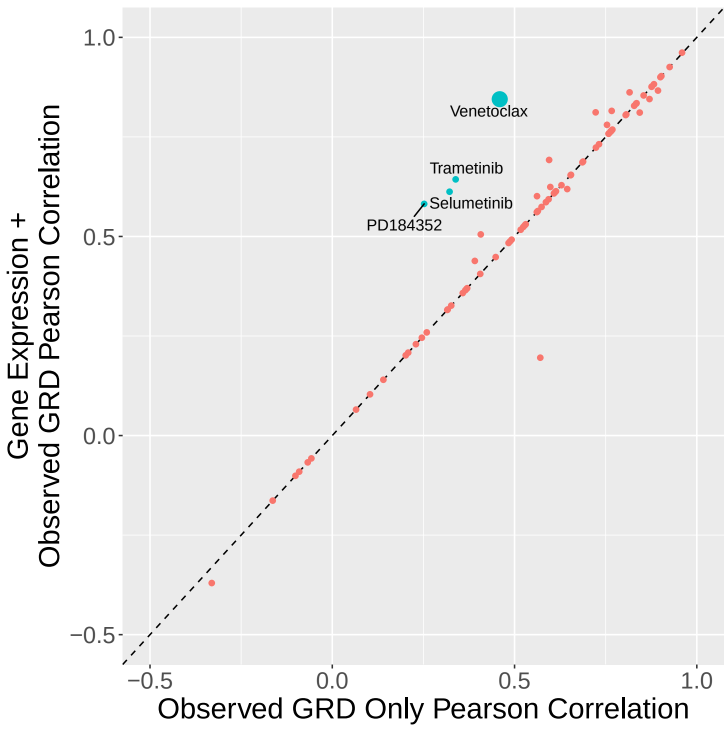

Supplementary Figure 33: Robust venetoclax resistance prediction is dependent on gene expression biomarkers and not general response across drugs. Performance (Pearson correlation) of ridge regression models trained on OHSU data and tested on FIMM data using only observed GRD as a predictor variable (x axis) or using gene expression and observed GRD (y axis). Each point corresponds to a drug ($n = 86$).

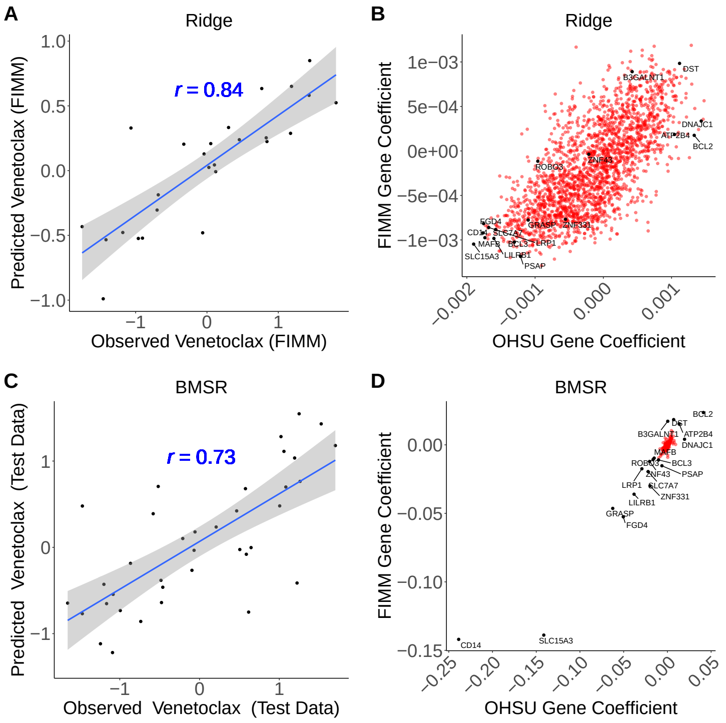

Supplementary Figure 34: Integrative analysis reveals monocyte-associated biomarkers predictive of venetoclax resistance. (A,C) Observed (x axis) versus model-predicted (y axis) venetoclax response. (A) Expression-based ridge regression model trained on ($n = 170$) OHSU samples and tested on ($n = 26$) FIMM samples. (C) Expression-based Bayesian regression model trained using five-fold cross validation on combined OHSU and FIMM datasets ($n = 159$) and tested on held-out fold yielding median performance across the five folds ($n = 37$). (B,D) Coefficients of genes ($n = 2,132$) in OHSU (x axis) or FIMM (y axis) datasets following (B) training of ridge regression model independently on both datasets or (D) training of Bayesian regression modeling simultaneously on both datasets ($n = 196$). r: Pearson correlation; dashed line: identity line; blue line: linear regression fit; gray shading: 95% confidence interval. Labeled genes were those having extremal (Stouffer's $p < 0.01$) combined coefficients across both datasets. Boxplot indicates median, lower and upper hinges (at first and third quartiles, respectively), lower whisker [at the least value at most $1.5 \times$ IQR (inter-quartile range or distance between first and third quartiles) below the lower hinge] and upper whisker (at the greatest value at most $1.5 \times$ IQR above the upper hinge).

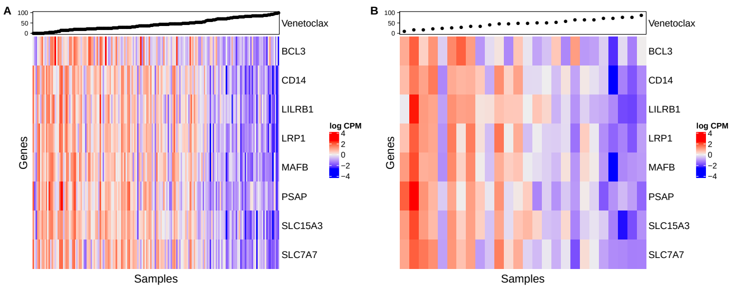

Supplementary Figure 35: Expression of monocyte-associated venetoclax biomarkers are consistently upregulated in venetoclax-resistant samples. Expression (standardized base-2 logarithm of counts per million, $\log_2$(CPM)] of BMSR-prioritized, monocyte-associated venetoclax biomarkers in (A) OHSU and (B) FIMM datasets. Samples are ordered according to venetoclax response (AUC).

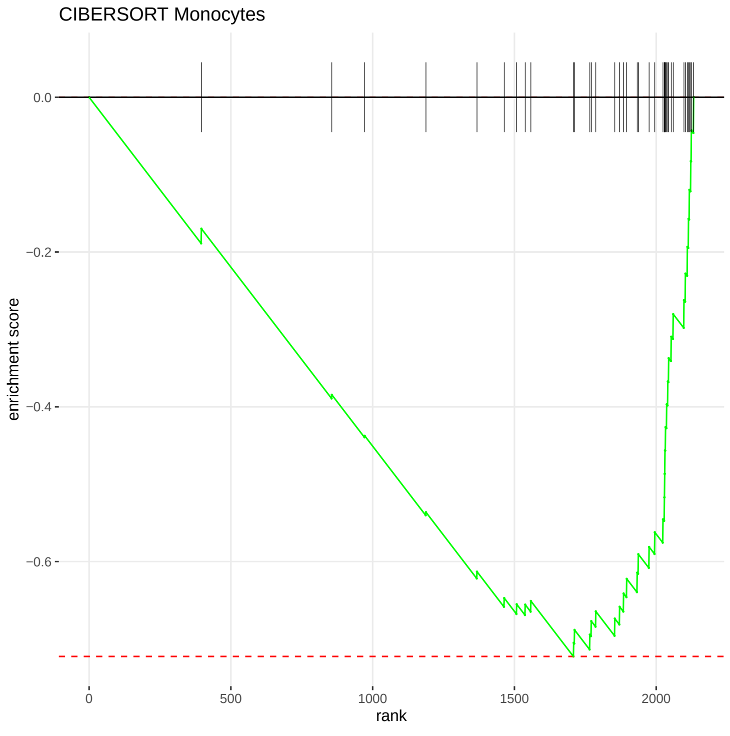

Supplementary Figure 36: Genes with expression correlated with venetoclax resistance are enriched for monocytic markers. Gene set enrichment analysis of genes ranked according to mean standardized ridge regression coefficients from models independently trained on OHSU and FIMM datasets.

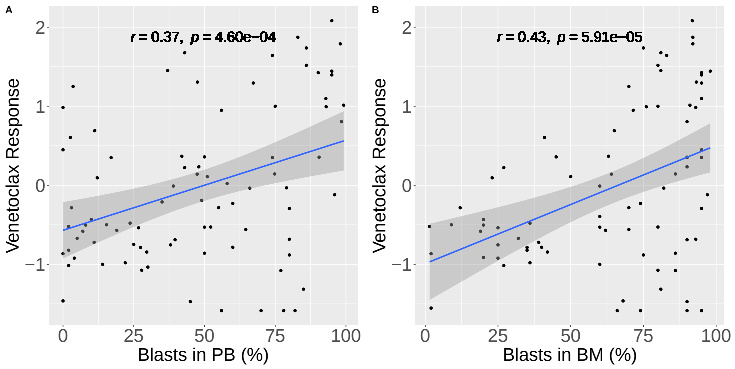

Supplementary Figure 37: Venetoclax response is correlated with blast fraction. Venetoclax response (AUC) versus percentage of blasts in (A) peripheral blood (PB) or (B) bone marrow (BM) in OHSU dataset. r: Pearson correlation; blue line: linear regression fit; gray shading: 95% confidence interval.

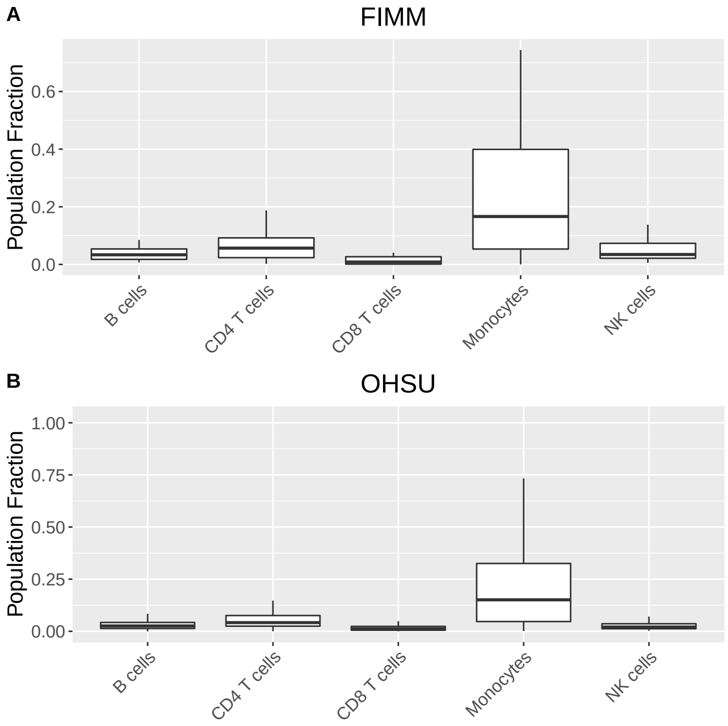

Supplementary Figure 38: Monocytic cell fractions are higher and more variable than lymphocyte fractions in *ex vivo* samples. CIBERSORT-derived fractions of monocytes and lymphocytes (B, CD4 T, CD8 T, and NK cells) in (A) FIMM and (B) OHSU. B cell fractions are the sum of naive and memory B and plasma cell fractions; CD4 T cell fractions are the sum of naive CD4 T, resting memory CD4 T, activated memory CD4 T, and regulatory T cell fractions; NK cell fractions are the sum of resting and activated NK cell fractions.

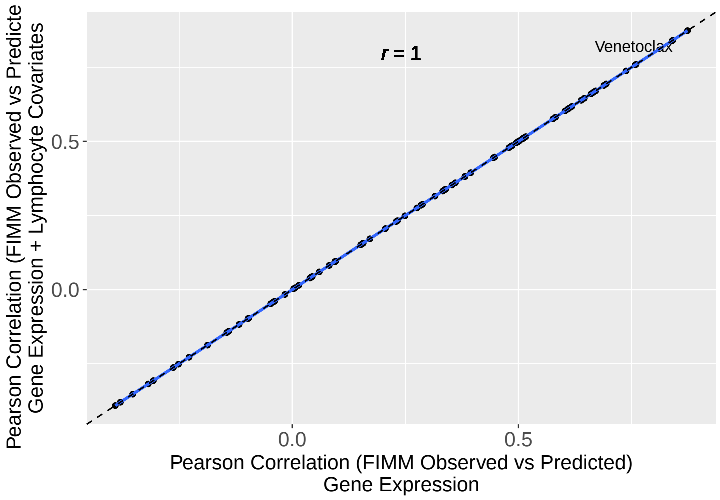

Supplementary Figure 39: Prediction model controlling for lymphocyte fraction is highly correlated with model that does not. Correlation of drug response observed in FIMM dataset relative to that predicted from OHSU training dataset using gene expression (x axis) versus using gene expression and lymphocyte fraction (y axis). Each point corresponds to a drug (n=87). r: Pearson correlation; blue line: linear regression fit; gray shading: 95% confidence interval.

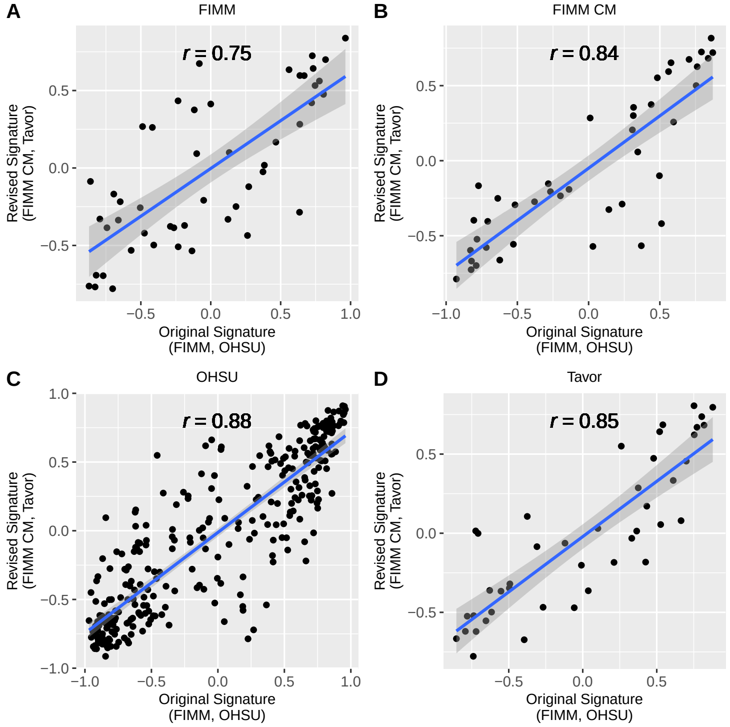

Supplementary Figure 40: BMSR-derived monocytic signature is stable across datasets jointly analyzed. Revised monocytic signature using genes inferred from joint BMSR analysis of FIMM (CM) and Tavor (y axis) versus original monocytic signature using genes inferred from joint BMSR analysis of FIMM and OHSU (x axis) in (A) FIMM, (B) FIMM (CM), (C) OHSU, and (D) Tavor datasets. Each point corresponds to one patient [FIMM: n=50; FIMM (CM): n=42; OHSU: n=313; Tavor: n=43]. r: Pearson correlation; blue line: linear regression fit; gray shading: 95% confidence interval.

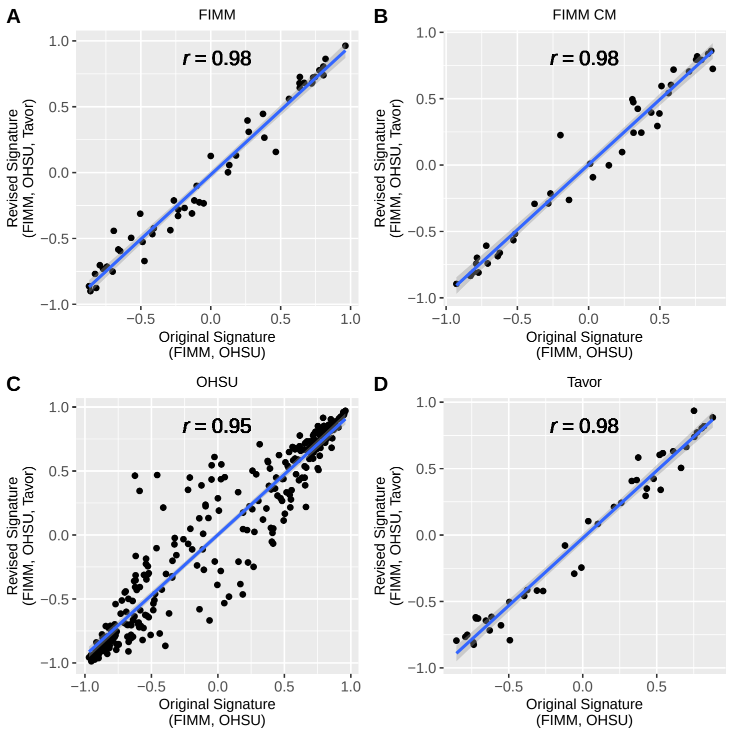

Supplementary Figure 41: BMSR-derived monocytic signature is stable across number of datasets jointly analyzed. Revised monocytic signature using genes inferred from joint BMSR analysis of FIMM, OHSU, and Tavor (y axis) versus original monocytic signature using genes inferred from joint BMSR analysis of FIMM and OHSU (x axis) in (A) FIMM, (B) FIMM (CM), (C) OHSU, and (D) Tavor datasets. Each point corresponds to one patient [FIMM: n=50; FIMM (CM): n=42; OHSU: n=313; Tavor: n=43]. r: Pearson correlation; blue line: linear regression fit; gray shading: 95% confidence interval.

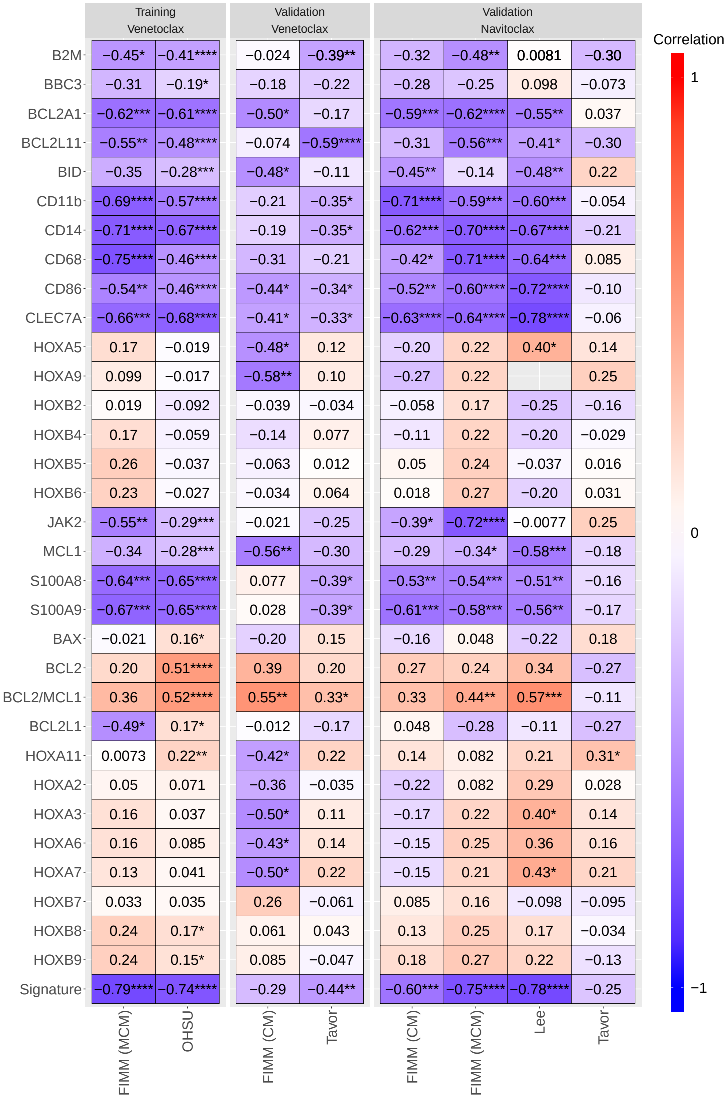

Supplementary Figure 42: Monocyte-associated genes and associated signature robustly predict resistance to BCL-2 inhibitors. Pearson correlation of response of indicated drug (venetoclax or navitoclax; top) versus expression of indicated gene or venetoclax monocyte signature ("Signature") across FIMM (MCM), OHSU, FIMM (CM), Lee, or Tavor datasets. Dataset / drug combinations are indicated as "Training" if they were used to derive the signature and biomarkers and "Validation" otherwise. *BCL2A1*, *CLEC7A*, and *CD14* have previously been nominated as biomarkers using the OHSU dataset (which should be considered a training dataset for these genes). *BCL2/MCL1* is the ratio of expression of *BCL2* and *MCL1*, i.e., the difference in their log expression. ****: $p < 0.0001$; ***: $p < 0.001$; **: $p < 0.01$; *: $p < 0.05$.

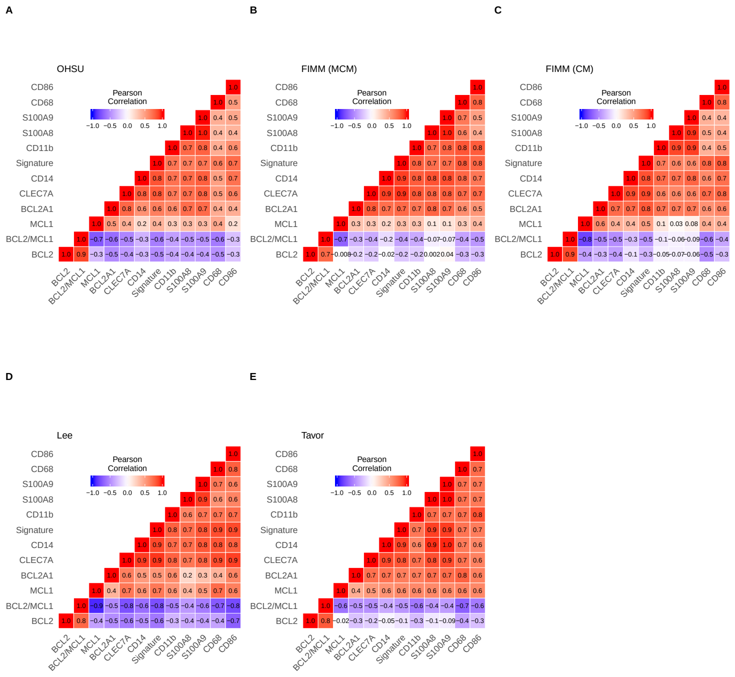

Supplementary Figure 43: Venetoclax biomarkers proposed in the literature have highly coordinated expression with monocytic signature. Pairwise pearson correlation of venetoclax biomarkers in the (A) OHSU, (B) FIMM, (C) FIMM CM, (D) Lee, and (E) Tavor datasets.

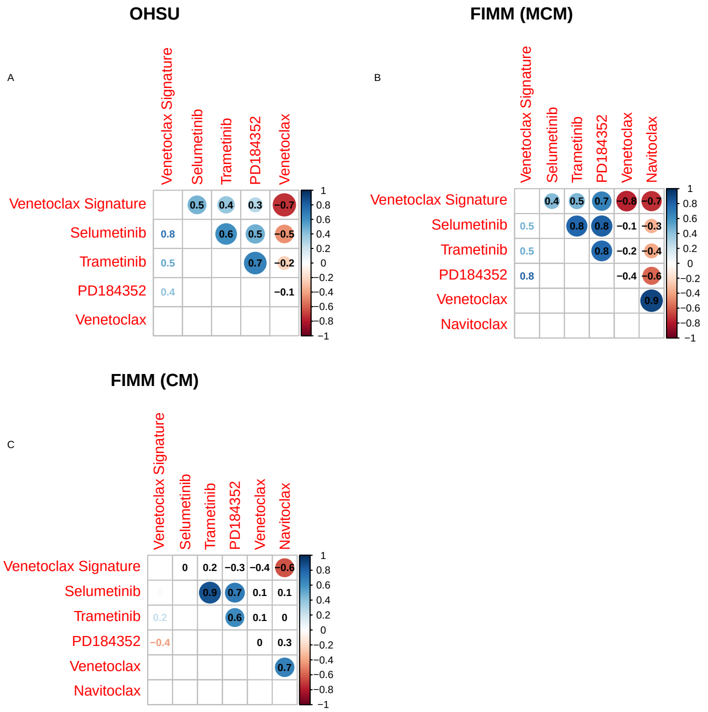

Supplementary Figure 44: Monocytic signature is correlated with MEK inhibitor response. Pairwise Pearson correlations of monocytic signature (Venetoclax Signature), BCL-2 inhibitor response (venetoclax and navitoclax), and MEK inhibitor response (selumetinib, trametinib, and PD184352) in the (A) OHSU, (B) FIMM, and (C) FIMM CM datasets. Above diagonal: raw Pearson correlations; absence of red or blue circle indicates correlation is not significant ($p > 0.05$). Below diagonal: Pearson correlation of MEK inhibitor m and monocytic signature normalized by Pearson correlation of m and best fit line of m versus other MEK inhibitors $m' \neq m$.

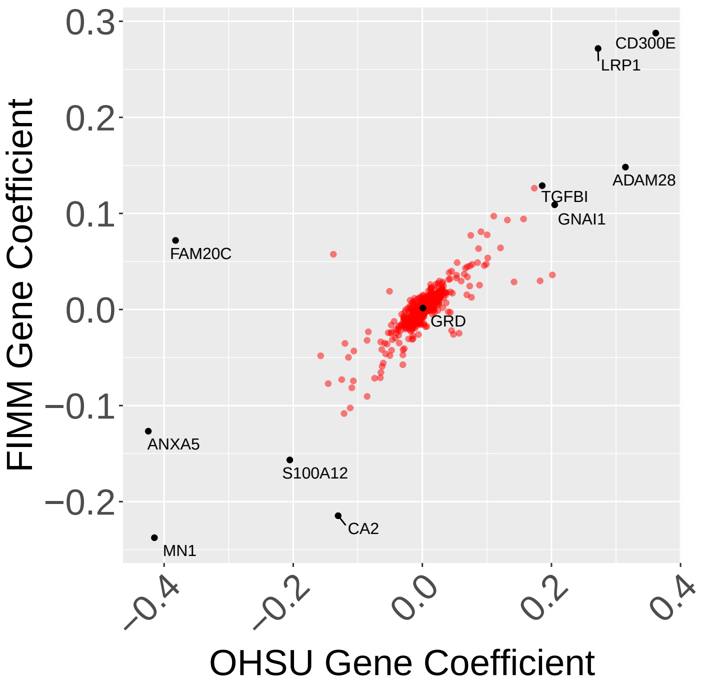

Supplementary Figure 45: Integrative analysis reveals monocyte-associated biomarkers predictive of MEK inhibitor response. Coefficients of genes (n=609) and observed GRD in OHSU (x axis) or FIMM (y axis) datasets following training of Bayesian regression modeling simultaneously on both datasets (n=259). Labels indicate observed GRD or the ten genes having the greatest absolute mean coefficient across both datasets.
